# Targeting cystine addiction to treat breast cancer lung metastasis

**DOI:** 10.1158/0008-5472.CAN-25-3883

**Published:** 2026-10-02

**Authors:** Josep Tarragó-Celada, Asad Mahmood, Yulia Panina, Sakshi Lalwani, Samantha Atkinson, Amy Spicer, Michael Kossifos, Weigang Cai, Ersa Gjelaj, Sharavan Vishaan Venkateswaran, Saidu Sesay, Izadora Furlani, Nathalie M. Legrave, Tegan Glimore, Richard Mitter, Jayanta Bordoloi, Avinash Ghanate, Vincen Wu, Zoltan Takats, Bin Yan, Alex Dexter, Rory Steven, Josephine Bunch, George Georgiou, Everett Stone, Míriam Tarrado-Castellarnau, James I. MacRae, Richard J. Burt, Mariia Yuneva

**Affiliations:** 1 Oncogenes and Tumour Metabolism Laboratory, The Francis Crick Institute, London NW1 1AT, UK; 2 Department of Immunology and Inflammation, Faculty of Medicine, Imperial College London, South Kensington Campus, London SW7 2AZ, UK; 3 Biological Research Facility, The Francis Crick Institute, London NW1 1AT, UK; 4 Metabolomics Platform, The Francis Crick Institute, London NW1 1AT, UK; 5 Metabolomics Platform, Luxembourg Institute of Health, L-1445 Strassen, Luxembourg; 6 Bioinformatics and Biostatistics Platform, The Francis Crick Institute, London NW1 1AT, UK; 7 In Vivo Imaging Platform, The Francis Crick Institute, London NW1 1AT, UK; 8 Department of Metabolism, Digestion and Reproduction, Faculty of Medicine, Imperial College London, South Kensington Campus, London SW7 2AZ, UK; 9 National Centre of Excellence in Mass Spectrometry Imaging, National Physical Laboratory, Hampton Rd, Teddington TW11 0LW, UK; 10 Department of Molecular Biosciences, The University of Texas at Austin, Austin, Texas 78712, USA; 11 Department of Biochemistry and Molecular Biomedicine and Institute of Biomedicine of Universitat de Barcelona, Faculty of Biology, Universitat de Barcelona, Barcelona 08028, Spain; 12 CIBER of Hepatic and Digestive Diseases (CIBEREHD), Institute of Health Carlos III (ISCIII), Madrid 28020, Spain; 13 University College London Hospital, London NW1 2BU, UK; 14 Calico Life Sciences LLC, South San Francisco, CA, USA 94080

**Keywords:** Keywords, Breast cancer, lung metastasis, glutathione, cyst(e)inase, radiotherapy

## Abstract

Metastatic disease remains a major cause of cancer-related mortality. Recent studies suggest that dissemination to other organs comes with metabolic changes that allow the metastasising cancer cells to adapt to new microenvironments. A deeper knowledge of these specific metabolic features and associated vulnerabilities could lead to the development of more effective therapies against metastasis. We used *in vivo* and *ex vivo* models of MYC-driven breast tumorigenesis to explore the key metabolic pathways that change when mammary gland tumour cells metastasise to the lung. By stable isotoperesolved metabolomics, mass spectrometry imaging and single-cell RNA sequencing we demonstrate that mammary gland tumour-derived lung metastases have increased synthesis of glutathione fuelled by increased cystine uptake. Our results uncover that metastatic cells rely heavily on the availability of extracellular cysteine or cystine, possibly due to downregulated intracellular cysteine synthesis through transsulfuration pathway. Based on this finding of cysteine/cystine addiction, we show that when combined with focal radiotherapy, the amino acid degrader cyst(e)inase can effectively reduce metastatic burden in the lungs. This novel combinatorial approach exploits a metabolic dependency that is unique to the metastatic cells, and acts as a sensitizer to radiotherapy-induced oxidative stress, offering a promising targeted strategy.

## Introduction

Female breast cancer is the second most frequent type of cancer worldwide, and the fourth most common in terms of mortality (1). The main cause of cancer-related deaths is metastatic disease, which currently lacks effective therapies (2). Recent studies suggest that changes in metabolism of tumour cells are required for cancer cells to survive and grow at a distant site (3). Glutathione (GSH) metabolism, a major antioxidant pathway in mammalian cells, has emerged as a pivotal player in maintaining redox homeostasis and protecting tumour cells from oxidative stress during metastatic spread (4). In addition to the initial discovery that antioxidant supplementation contributes to greater metastatic incidence in a melanoma model (5) the upregulation of the antioxidant-response transcription factor NRF2, along with GSH-related genes, have also been observed in *in vivo* models of breast cancer metastasis (6). Additionally, enhanced GSH synthesis has been recently found to be a key requirement for the successful metastasis of breast cancer to the lung and liver (7), GSH is a tripeptide synthesised from glutamate, glycine, and cysteine, amino acids, with cysteine availability as the rate-limiting step in its production (8). Cysteine can be obtained intracellularly through *de novo* synthesis by the transsulfuration (TS) pathway, or from extracellular cystine, which is transported into cells by the xCT amino acid transporter. While extracellular cystine is the primary source for glutathione in cancer cells (9), this reliance is very context-dependent (10). The activity of xCT transporter and the extracellular uptake of cystine have been shown to be important for metastatic spread in several types of cancers (11–13). However, the contribution of the TS pathway to GSH production during the metastatic process remains unclear.

Amplification of the cMYC oncogene is frequently observed in metastatic breast cancer(14), and is known to promote cell proliferation and metabolic reprogramming (15), including GSH synthesis as well as a characteristic of MYC-driven tumours (16). Herein, we use a cMYC-induced mammary gland cancer mouse model to evaluate metabolic differences between primary tumours and lung metastases. Our analyses revealed that increased levels and synthesis of GSH are major metabolic hallmarks of metastasis. We identified extracellular cystine as the main source fuelling the increased levels of GSH in lung metastases. Moreover, we determined that the metastatic cells are sensitive to cysteine/cystine deprivation and therefore addicted to these amino acids possibly due to suppressed activity of the TS pathway. Finally, we demonstrated that targeting the cysteine/cystine addiction of metastatic cells by treating tumour-bearing animals with the amino acid degrader cyst(e)inase (17) in combination with focal radiotherapy significantly reduces the lung metastatic burden *in vivo*. Our findings highlight a metabolic vulnerability in metastatic breast cancer and suggest an effective therapeutic strategy against breast cancer metastasis.

## Materials and Methods

### Mouse models, cell isolation and propagation, and tumour induction by injection

Wild-type FVB/NJ (Francis Crick Institute in-house colonies, RRID: IMSR_JAX:000664), and MMTV-MYC (Tg(MMETV-MYC)141-3Led) female mice, originally acquired from the Jackson Laboratory (RRID:MGI:2447500) were used in the study.

Mammary gland tumour cells Myc310 were originally established and generated previously in the laboratory from a transgenic MMTV-cMYC mouse which had multiple tumours. When injected into the mammary fat pad, Myc310 cells generated visible lung metastasis in 4-5 weeks. To generate a metastatic cell line that comes from the lung, LM1 and its biological replicates, whole lungs were isolated after 4-5 weeks of Myc310 cell injection into the fat pad. The lungs were cut into small chunks using sterile blades and transferred to 20 mL of collagenase buffer (1 mg/mL of collagenase, Thermo Fisher Scientific, 17104019, in DMEM:F12 Life Technologies 21331-046 with 1% BSA Gibco 10270, 10 mM HEPES Gibco 15630-056, 2 mM glutamine Gibco 25030-024, 5 μg/mL insulin Merck I1882, 1 μg/mL hydrocortisone Merck H0888, and 100 μg/mL of penicillin/streptomycin Gibco 15140-122), and shaken for 1 h at 37°C, 200 rpm. The tissue suspension was filtered through 70 μm filter, resuspended in TAC buffer (170 mM Tris, 150 mM ammonium chloride, pH 7.4), and incubated at 37°C for 1 h in order to lyse any red blood cells. The cell suspension was then spun down and resuspended in media for cell propagation (see Cell culture section).

For mammary fat pad cell injections, cells were grown in the media (see Cell culture and media section) and tested for mycoplasma. After detachment by trypsin (Thermo Fisher Scientific, 25-200-056), cells were centrifuged, washed twice with PBS, and resuspended in 50:50 PBS:Matrigel (Corning, 356230) on ice. 50 ul of cell suspension (50,000 cells/50 μL) were injected under the fourth inguinal mammary fat pad of 6-8 week old virgin FVB/NJ mice, ensuring a subcutaneous injection directed just below the nipple. Tumours were detected after approximately 2 weeks, measured twice a week from then, and at 4 weeks, mice were closely monitored for signs of lung metastatic burden. Lungs and tumours were immediately excised after mice were euthanised by isoflurane, flash frozen in liquid nitrogen and stored at -80°C. If half of each tissue samples was also fixed, it was incubated in 10% neutral buffered formalin for 24h at 4 °C, then transferred to 70% ethanol for 2-4 weeks before paraffin embedding.

All mouse procedures followed the United Kingdom Home Office regulations and were approved by the Animal Welfare and Ethical Review panel of The Francis Crick Institute, and performed under the Establishment license number X49D044E1, and the Project license number PP3464389.

### Cell culture

All mammary gland tumour mouse cell lines were cultured in DMEM:F12 1:1 (no glutamine, Gibco 21331-020), 10% FBS (Gibco 10270-106), 2 mM Glutamine (Gibco, 25030081), 1% P/S (Gibco, 15140122), 1% HEPES (Gibco 15630-080), 0.1% Insulin (Merck, I882), 0.1% Hydrocortisone (Merck, H0888), 0.01% EGF (Gibco, PMG8043). At 37°C and 5% CO_2_. In-house cell lines used in this publication are: MYAZ16.1e cells, isolated form a mammary tumour of an MMTV-cMYC mouse, and its biological replicates MYAZ12.3i and MYAZ6.6g; Myc310 cells, isolated from a mammary tumour of an MMTV-MYC mouse that had multiple tumours; LM1 cells, isolated from the lungs of mice bearing mammary gland tumours induced by mammary fat pad injection of Myc310 cell, and its biological replicates LM2, and LM3; LM1-LM cells, isolated from the lungs after LM1 cell injection in the mammary fat pad, and its biological replicates LM1-LM, LM2-LM, and LM3-LM. 4T1 (RRID: CVCL_0125) and 67NR (RRID: CVCL_9723) mouse mammary gland cancer cells have been published previously (24) and obtained from The Francis Crick Institute’s Cell Services platform.

shCBS lentiviruses were acquired from Santa Cruz Biotechnologies (sc-60335-V) and empty vector lentiviral particles (sc-108080) were used to infect MYAZ16.1e cells to perform stable CBS knockdowns according to the manufacturer’s instructions. Briefly, cells were seeded in 6 well plates at 30% confluence (70,000 cells), and 200 μL of the virus mix containing 2 × 10^5^ infectious units of virus was added, together with polybrene (at 8 μg/mL of final concentration). Cells were then incubated for 48h under standard culture conditions, and virus-containing medium was replaced by fresh medium. Cells were then used for subsequent experiments.

### Desorption electrospray ionisation mass spectrometry imaging (DESI-MSI)

Lungs and tumours were immediately excised after mice were euthanised by isoflurane, flash frozen in liquid and stored at -80°C. Fresh frozen tumour and lung samples were cryosectioned onto superfrost slides for DESI-MSI analysis. 10 μm sections were made using a Leica CM3050s cryostat. Adjacent sections were stained with H&E to confirm the presence of metastases in lung sections. Each frozen tissue sample was fixed onto a cryostat chuck using pre-cooled HPLC-water, taking care not to allow any tissue thawing. The cryostat temperature was kept at -22°C for lung sectioning, and -16°C for tumour sectioning. Prepared slides were dried using a nitrogen sprayer, and immediately vacuum-packed in individual slide containers, stored at -70°C until analysis to prevent any thermal degradation. Before the analysis sample slides were thawed at room temperature before breaking the vacuum seal to prevent moisture accumulation on a sample surface. DESI-MSI was performed using the Xevo™ G2-XS QToF mass spectrometer (Waters). DESI Imaging parameters were the following: Spray voltage 4.5 kV, nitrogen gas pressure 5 bar, solvent composition 95% MeOH in HPLC-grade water, solvent flow rate 1.5 μL/min, sprayer to inlet capillary distance 3 mm, sprayer to surface distance 0.5 mm, sprayer impact angle α 75°, collection angle β 10°, mass range 50 – 1000 m/*z*, scan rate 1 scan/sec, acquisition speed 100 μm/sec, mass resolving power 10,000 at 200 m/*z*, pixel diameter 100 μm. Following DESI-MSI, samples were re-vacuum packed to preserve tissue morphology, and stored at -70°C. For post H&E staining, samples were rehydrated in decreasing ethanol concentrations prior to fixation. DESI-MSI data analysis was performed using the HDImaging software (Waters), the MATLAB-based MSI data analysis software developed at the National Physical Laboratory, SpectralAnalysis (43), and an in-house DESI-MSI data visualisation script (https://clvd1-ws-u-p-58.thecrick.org/MIMA/e6194c9e457c1/). DESI-MSI ion annotation was performed using the CEUMass Mediator WCGNA analysis as reported in (44).

In order to quantify MSI images, data were first reduced to three dimensions using a neural network t-SNE approach (45) and visualised by RGB color-coding of each pixel by varying the red, green and blue intensity linearly on the three independent axes (46). Common features between these images and the H&E images were manually selected using the Matlab “*cpselect*” function. These registration points were then used to align the common features in these images using the Matlab “*cp2tform*” and “*imtransform”* functions. The Manual annotations from the H&E images were then converted into binary masks and transformed using the same approach. The intensities from all pixels for each of these registered regions were then extracted and plotted as boxplots using the Matlab “*boxplot”* function.

### Immunohistochemistry and H&E staining

Tissues were fixed in 10% neutral formalin solution for 24 h at 4°C, and changed to 70% ethanol at 4°C during a maximum of 4 weeks. Tissue were then paraffin-embedded, and slides were mounted by cutting them at 3 μm. Haematoxylin & eosin (H&E) staining was performed using a Tissue-Tek Prisma Plus Automated Slide Stainer & Coverslipper (Sakura Finetek). After deparaffination and hydration, slides were stained with haematoxylin for 90 seconds, and eosin for 2 minutes, with water and acid alcohol in between, and then dehydrated with increasing ethanol concentrations and xylene. Slides were then coverslipped using dibutyl phthalate polystyrene xylene (DPX) mounting media (Sigma). For immunohistochemistry (IHC), a BenchMark ULTRA Automated IHC/ISH slide staining system (Roche) was used. Image analysis and quantification of metastasis or positive cells were performed using QuPath software.

### Single-cell RNA sequencing

LM1 cells were injected into the mammary fat pads of 6-8 week old FVB/NJ female mice. Primary tumours and lung metastases were harvested after 4 weeks and finely minced with blades. The tissue fragments were dissociated in 2 mL of RPMI medium supplemented with 0.3 mg/mL Liberase (Roche) and 1 μg/mL DNase1 and incubated at 37°C for 45 minutes with intermittent vortexing. To halt the enzymatic reaction, 10 mL of wash buffer (PBS containing 3% FBS and 2 mM EDTA) was added to the dissociated lung tissue. The resulting cell suspension was gently mixed, filtered through a 70 μm cell strainer, and centrifuged at 300 × *g* for 5 minutes. After an additional wash, cell pellets were resuspended in 2 mL of red blood cell lysis buffer (Merck) and incubated at 37°C for 3 minutes. The lysis was stopped by adding 20 mL of wash buffer, followed by filtration through a 40 μm strainer and another centrifugation at 300 × *g* for 5 minutes. The final cell pellets from both tumour and lung samples were resuspended in PBS with 1% BSA. For viability quality control, 150 μL of the suspension (at 1000 cells/μL) was transferred to LoBind Tubes (Eppendorf, 022431021) and assessed using the LUNA-FX7 system. Only samples with a viability greater than 80% proceeded to emulsion generation and library preparation, with sequencing libraries constructed from 10,000 cells per sample. Tumour samples were processed individually, while cells from two lungs were pooled for each lung sample.

Sample-level count data were generated from fastq files using 10x Genomics Cell Ranger 7.1.0, aligned to the 10X mouse reference transcriptome (refdata-gex-mm10-2020-A), with reads mapped to both intronic and exonic regions. The resulting filtered feature-barcode matrices served as input for downstream analysis in R using the Seurat package, applying default settings unless otherwise specified. Count matrices were normalised on a per-cell basis using centered log ratio (CLR) transformation [Seurat::NormalizeData(normalization.method = “CLR”, margin = 2)]. The 2,000 most variable genes, exhibiting the highest cell-to-cell variation, were identified [Seurat::FindVariableFeatures(selection.method = “vst”, nfeatures = 2000)], and the data were scaled and centred across all features [Seurat::ScaleData] to ensure uniform mean expression and variance, thereby preventing highly expressed genes from skewing subsequent analyses. Principal component analysis (PCA) was performed on these variable genes to assess dataset dimensionality, followed by UMAP for nonlinear dimensionality reduction using the first 30 principal components, enabling similar cells to cluster together in low-dimensional space. A k-nearest neighbours (KNN) graph was constructed based on Euclidean distances in PCA space, with edge weights refined by Jaccard similarity [Seurat::FindNeighbors(dims = 50, k.param = 20, compute.SNN = TRUE)], and used for Louvain algorithm clustering [Seurat::FindClusters(resolution=0.5)]. Cell cycle heterogeneity was evaluated by scoring each cell according to canonical G2/M and S phase marker genes [Seurat::CellCycleScoring], while potential doublets were flagged using the DoubletFinder package [NO_PRINTED_FORM], assuming a doublet rate of 7.5%.

After processing each sample separately, integration features—genes consistently variable across datasets—were selected [Seurat::SelectIntegrationFeatures]. Integration anchors, representing biologically similar cell pairs across datasets, were identified using reciprocal PCA focused on these features. These anchors enabled the integration of all sample datasets into a single object via Seurat::IntegrateData function. The integrated data were then centered, scaled, and subjected to PCA, UMAP, KNN graph construction, and Louvain clustering as described for the individual samples (see above). Automated cell-type annotation was performed on the raw count data by comparing gene signatures to the Mouse Cell Atlas using the scMCA package (v0.2.0). Initial unsupervised cluster identities were assigned based on the predominant cell-level annotation within each cluster and further refined through manual inspection of known marker genes.

A pseudobulk approach was used to test for differential gene expression between primary tumour cells and tumour metastatic cells within clusters. Briefly, genes present in <5 cells across the dataset were discarded. The Bioconductor package glmGamPoi was used to aggregate counts, fit gene-wise Gamma-Poisson generalised linear models [glmGamPoi::glm_gp(size_factors = “ratio”)] and conduct quasi-likelihood ratio tests between cell-types. The Benjamini-Hochberg method was used to control the false discovery rate due to multiple testing. A threshold for significance was set as fdr<0.05. Lists of significantly changing genes were further split based on their direction of change and used as input for clusterProfiler’s compareCluster function to assess association with gene sets of known biological function from Reactome and GOBP collections, against a background of the mouse genome. Input gene lists containing fewer than 30 genes were not considered for testing.

### mRNA sequencing from cell lines

Cells were seeded at 250,000 cells/well in 6-well plates and cultured as described in The cell culture section. The following cell lines were seeded: MYAZ16.1e, MYAZ12.3i, MYAZ6.6g, Myc310, LM1, LM2, LM3, LM1-LM, LM2-LM, LM3-LM. After 24h, media was changed to the control or cysteine/cystine free media for 6 h (only for MYAZ16.1e, Myc310, LM1, and LM1-LM). Then, RNA extraction was performed according to RNAeasy kit (QIAGEN 74104), including DNA digestion with DNAse I set (QIAGEN 79254). Three samples per a cell line and a condition were submitted for RNA sequencing.

RNA quality was determined by electrophoresis in 4200 TapeStation system (Agilent). mRNA libraries were prepared using the NEBNext Ultra II Directional PolyA kit (Illumina). Paired-end sequencing was then performed on the NovaSeq 6000 (Illumina) with a read length of 100bp and to a minimum read count 25 million per sample.

Fastq files were run through alignment and read quantification via the nf-core RNAseq pipeline (nf-core version 3.10.1, Nextflow version 22.10.3, using STAR and RSEM options). Two different designs were used in combination with the default settings of DESeq2 to test differential expression. The “metastatic progression” group of comparisons used samples with a MYAZ genotype in the control condition, applied a model considering subtype only, and compared each cell subtype. The “cysteine/cystine dependency” group included MYAZ16_1e, Myc310, LM1, and LM_LM1 genotypes, and the influence of subtype, condition, and their interaction were modelled. Comparisons were made between the cysteine/cystine deprivation condition against the subtype-matched control. Ensembl gene IDs were associated with their common names using AnnotationDbi.

Gene Set Enrichment Analyses (GSEA) were performed against the Curated Gene Sets MSigDB Collection, against terms in the GTRD subset of the Transcription Factor Targets prediction gene sets, and also in a subset of the Canonical pathways collection comprising of Reactome, WikiPathways, and KEGG ontologies. These were downloaded from MSigDB via msigdbr (version 7.5.1) for mouse ontologies. Differential enrichment was tested via clusterProfiler (version 4.6.2), using gene lists per contrast ordered by decreasing ashr-shrunk (version 2.2-63) Log2 Fold Change (LFC). An adjusted *p* value cutoff of 0.05 was used to determine significant enrichment (Benjamini-Hochberg method).

### Stable isotope-resolved metabolomics *in vivo*

All stable isotope-resolved metabolomics (SIRM) experiments *in vivo* were performed by infusion of isoflurane-anaesthetised animals through a tail vein catheter using an Aladdin AL-1000 pump (World Precision Instruments), as previously described (47). For [U-^13^C]glutamine, mice first received a bolus of 0.187 mg/g of body weight, followed by a 0.005 mg/g of body weight per minute for 3 h at 0.15 mL/h. For [U-^13^C^15^N]cystine, mice received an infusion of 1.438 ug/g of body weight per minute for 3 h at 0.15 mL/h. For [U-^13^C]serine, mice received first a bolus of 0.1275 mg/g of body weight, followed by a 2.875 ug/g of body weight per minute for 3 h at 0.15 mL/h. At the end of the infusion, mice were culled and tissues were collected and frozen in liquid nitrogen. Where indicated metastatic lesions were carved out of the metastatic lung based on their opac white colour. Blood was collected through cardiac puncture, incubated for 10 minutes at 4°C and centrifuged at 10,000 g for 10 minutes. Supernatant was collected and immediately frozen in liquid nitrogen. Frozen tissues were ground by mortar and pestle in liquid nitrogen and freeze dried using FreeZone Labconco Freeze Dryer.

### Stable isotope-resolved metabolomics *in vitro*

For *in vitro* SIRM experiments, [U-^13^C^15^N]cystine incubations were done for 6 h and 24 h after seeding 100,000 cells / well in 6-well plates, 2 mL of media, changing the media to either labelled or unlabelled media at the beginning of the incubation. [U-^13^C]serine incubations were done for either 24 h or 52 h, 24 h after seeding 60,000 cells / well in a 6-well plate, with 2 mL of media, changing the media to either labelled or unlabelled media at the beginning of the incubation. At the end of the incubation, metabolite extraction was directly performed.

### Metabolite extraction and quantification by UPLC-MS analysis

Polar metabolite extraction from tissue and serum samples was performed based on methods previously described (48). 300 μL of extraction HPLC-grade solvents (methanol/acetonitrile/water, 5:3:2 (v/v)), was added to approximately 3 mg of dried tissue powder. An internal standard of [U-^13^C,^15^N]valine was added to each sample at a final concentration of 5 μM. Each sample was then vortexed, sonicated three times (8 minutes each), and centrifuged at 21,000 × *g*, 4°C for 10 min. The supernatant was then moved to a new Eppendorf tube, and the pellet was once again re-extracted using 150 μL of the same extraction solvents. The samples were vortexed, sonicated, and centrifuged again. The supernatants were pooled for a final extract of 450 μL, of which 100 μL was used for UPLC-MS analysis, together with pooled biological quality controls (PBQCs – a representative pool sample, consisting of 10 μL of each sample), and metabolite standards.

For serum samples, 5 μL of serum was added to 15 μL cold HPLC-grade methanol in a 500 μL Eppendorf tube, which was vortexed and centrifuged at 21,000 × *g*, 4°C for 10 min. The supernatant was then added to a mix of 135 μL of methanol, 150 μL of water (including [U-^13^C,^15^N]valine for a 5 μM final concentration), and 50 μL of chloroform. Samples were vortexed and centrifuged 21,000 × *g*, 4°C for 10 minutes, and the polar phase was used for HPLC-MS analysis, together with PBQCs and metabolite standards.

Polar metabolite HPLC-MS analysis was performed on a Vanquish LC coupled to a Q-Exactive Plus Mass Spectrometer (both Thermo Scientific). Chromatographic separation was performed on a SeQuant® ZIC®pHILIC column (Merck, 5 μm particle size, polymeric, 150 × 4.6 mm). Injection volume was 10 μL, the column oven was maintained at 25°C, and the autosampler temperature was maintained at 4°C. Chromatographic separation was achieved using gradient elution at a constant flow rate of 300 μL/min over a total run time of 25 min. An initial mobile phase of 80% solvent B was held for 2 min, then it decreased from 80 to 5% over 15 minutes, holding at 5% for 3 minutes and finally re-equilibrating to 80% B for 5 minutes. Solvent A was 20 mM ammonium carbonate solution in water and solvent B was acetonitrile. Column eluant was introduced to the MS through a HESI II probe. MS was performed with polarity switching with parameters as follows: spray voltage 3.5 and 3.2 kV for both positive and negative modes, respectively; probe temperature 320°C; sheath and auxiliary gases were 30 and 5 arbitrary units, respectively; and full scan range: 80-1000 m/*z* with settings of AGC target and resolution as balanced and high (1e^6^ and 70,000), respectively. Data were recorded using Xcalibur 4.2.47 software (Thermo Scientific). Mass calibration was performed for both polarities before analysis using the standard Calmix solution (Thermo Scientific). To enhance calibration stability, lock-mass correction was also applied to each analytical run using ubiquitous low-mass contaminants.

Metabolites were identified by the comparison of the accurate mass, fragmentation pattern, and retention time to authentic chemical standards run in the same batch using TraceFinder 4.1 EFS software (Thermo Scientific). Quantification and label incorporation was calculated after natural isotope abundance correction using an in-house LC-MS data analysis script, (https://avicrick.shinyapps.io/MSIso/). For each metabolite, the area under the curve was normalised to the area under the curve of the internal standard [U-^13^C,^15^N]valine and by dried sample weight measured prior to metabolite extraction. For FE calculations, M+n/ΣM formula was used, where M is the intensity of each isotope of the molecule (i.e., M+0, M+1, M+2, etc).

### Metabolite extraction and quantification by UPLC-MS analysis for cysteine/cystine and oxidised/reduced glutathione species

The extraction of the polar fraction from tissue samples was performed as described above with an exception of the last step when a polar phase was dried on a rotary vacuum concentrator (Christ AVC 2-33 CD plus) and resuspending in 30 μL of extraction solvents (methanol/acetonitrile/water, 5:3:2 (v/v)). Extraction from serum samples was performed exactly as described in the previous section. To extract metabolites from cell samples (6-well plate), cells were washed twice with 1 mL ice-cold PBS, 50 μL of extraction HPLC-grade solvents (methanol/acetonitrile/water, 5:3:2 (v:v:v)) was added to the well, cells were recovered using a cell scraper, transferred to a chilled 1.5 mL Eppendorf, vortexed (3 pulses of 5 seconds each), and centrifuged at 21,000 × *g* and 4°C for 10 minutes. Supernatant was collected and submitted for UPLC-MS analysis.

Metabolite analysis was performed using an Agilent Infinity Poroshell 120 HILIC (2.1 × 150 mm, 1.7 µm) for chromatographic separation. The following LC parameters were used: injection volume, 5 μL; column oven temperature, 25°C, autosampler temperature, 4°C. Chromatographic separation was achieved using gradient elution at a constant flow rate of 300 μL/min over a total run time of 19 minutes. An initial mobile phase of 80% solvent B was held for 2 minutes, decreased to 5% over 10 minutes, holding at 5% for 2 minutes and finally re-equilibrating to 80% B for 5 minutes. Solvent A was 0.01 % of formic acid, and solvent B was acetonitrile.

MS was performed in positive ion mode with the following parameters: spray voltage 3.5 kV; probe temperature 320°C; sheath and auxiliary gases were 30 and 5 arbitrary units, respectively; full scan range: 80–1000 m/*z* with settings of AGC target and resolution as balanced and high (1e^6^ and 70,000), respectively. Selected ion monitoring (SIM) was performed to detect cysteine, cystine, reduced and oxidised glutathione, and their respective isotopologues. The parameters used for SIM were: resolution 70,000; AGC target 3e^6^; maximum IT 200 ms; isolation window 0.4 m/*z*; spectra were centroid; collision energies were set individually in HCD (high-energy collisional dissociation) mode. Cystine, cysteine, reduced and oxidized glutathione standards were prepared and spiked into individual sample matrices of interest. Retention times were corrected according to the matrix type. Metabolites were identified by comparison of accurate mass, fragmentation, and retention time to authentic chemical standards run in the same batch using TraceFinder 4.1 EFS software (Thermo Scientific).

### RT-qPCR

RNA was isolated either from cells a pellet of 1 M cells or 3g of dry tissue using RNAeasy kit (QIAGEN 74104), including DNA digestion with DNAse I set (QIAGEN 79254). After RNA quantification by Nanodrop (Thermo Fisher Scientific 2000C), RNA was converted to cDNA by adding 5 μg RNA to a PCR tube with 1 μL of random primers (0.5 μM/μL), 1 μL of dNTP mix (10 mM, Thermoscientific R0192), 1 μL of DTT (0.1M), 0.5 μL of RNase OUT (Invitrogen 10777/019), 0.5 μL of Superscript III RT (Invitrogen 18080-044), and 4 μL of 5X first strand buffer (Invitrogen Y02321), up to 20 μL with DEPC-treated H_2_O, which was incubated for 45 minutes at 50°C and inactivated for 15 minutes at 70°C. cDNA was then quantified using Nanodrop and 2 μL of cDNA (150 ng/μL) were loaded into a 384-well plate with 5 μL of SYBR Green PCR Master Mix (Thermo Scientific 4367659), 2 μL of DEPC-treated H_2_O, and 0.5 μL of each reverse and forward primer.

Primers used were:

mouse *Gclc* Forward: 5’-ATGTGGACACCCGATGCAGTATT-3’;

mouse *Gclc* Reverse: 5’-TGTCTTGCTTGTAGTCAGGATGGTTT-3’;

mouse *Gsr* Forward: 5’-GCCTTTACCCCGATGTATCACGCTGTG-3’;

mouse *Gsr* Reverse: 5’-TGTGAATGCCAACCACCTTTTCCTCTTT-3’;

mouse *Cbs* Forward: 5’-GGGACAAGGATCGAGTCTGGA-3’;

mouse *Cbs* Reverse: 5’-AGCACTGTGTGATAATGTGGG-3’;

mouse *Cth* Forward: 5’-TTCCTGCCTAGTTTCCAGCAT-3’;

mouse *Cth* Reverse: 5’-GGAAGTCCTGCTTAAATGTGGTG-3’;

mouse *Gss* Forward: 5’-CAAAGCAGGCCATAGACAGGG-3’;

mouse *Gss* Reverse: 5’-AAAAGCGTGAATGGGGCATAC-3’;

mouse *Gpx1* Forward: 5’-AGTCCACCGTGTATGCCTTCT-3’;

mouse *Gpx1* Reverse: 5’-GAGACGCGACATTCTCAATGA-3’;

mouse *Gpx3* Forward: 5’-CCTTTTAAGCAGTATGCAGGCA-3’;

mouse *Gpx3* Reverse: 5’-CAAGCCAAATGGCCCAAGTT-3’;

mouse *Gpx4* Forward: 5’-GATGGAGCCCATTCCTGAACC-3’;

mouse *Gpx4* Reverse: 5’-CCCTGTACTTATCCAGGCAGA-3’;

mouse *Slc7a11* Forward: 5’-TGGGTGGAACTGCTCGTAAT-3’;

mouse *Slc7a11* Reverse: 5’-AGGATGTAGCGTCCAAATGC-3’;

mouse *Actb* Forward: 5’-GTGACGTTGACATCCGTAAAGA-3’;

mouse *Actb* Reverse: 5’-GCCGGACTCATCGTACTCC-3’.

### Western blotting

50,000 cells were seeded in 6-well plates. At indicated times they washed twice with PBS and incubated for 20 minutes in RIPA buffer (50 mM Tris pH 8.0, 150 mM NaCl, 1% Triton X-100, 0.5% Sodium deoxycholate, 0.1% sodium dodecyl sulphate, 1:100 protease inhibitor cocktail (Thermo Fisher Scientific, 78429), 1:100 of 1M NaF, and 1:200 of 200 mM Na_3_VO_4_ as phosphatase inhibitors). After scraping and sonicating (3 times for 15 seconds each time with ultrasonic titanium probe at 950 watts of ultrasonic outpuc, 145 μm of vibration amplitude), samples were centrifuged at 12,000 × *g*, 4°C for 20 minutes. Protein content of the supernatants was quantified by BCA method, and loading buffer (Bio-Rad, 1610747 + 1:10 of β-mercaptoethanol Bio-Rad 1610710) was added to the samples before electrophoresis by SDS-PAGE. Afterwards, proteins were transferred to nitrocellulose membranes and blocked with 5% skim milk in PBS with 0.1% Tween-20 during 1 h. Then, membranes were incubated with an indicated antibodies overnight, and finally the suitable HRP-labelled secondary antibody was applied before and after 3 washes of 10 minutes each with PBS 0.1% Tween-20. Luminescent image of the membranes was taken after applying Amersham Cytiva RPN2236 reagent and using an automatic developer (Amersham ImageQuant 800). We used the following primary antibodies: CBS (Cell Signalling, 14782S, 1:500, RRID: AB_2798609), CTH (Cell signalling, 19689, 1:1000, RRID: AB_2798824), xCT (Abcam, ab175186, 1:1000, RRID: AB_2722749), GCLC (ThermoFisher, PA519702, 1:900, RRID: AB_11008589), GPX1 (Abcam, ab22604, 1:1000, RRID: AB_2112120), GPX3 (Abcam, ab256470, 1:1000, RRID: AB_3718666), GPX4 (Invitrogen, MA532827, 1:1000, RRID: AB_2810103), GSR (Abcam, ab93439, 1:1000, RRID: AB_10712168), VCL (ThermoFisher, 700062, 1:1000, RRID: AB_2532280). The secondary antibodies used: anti-rabbit (Invitrogen, 65-6120, 1:5000, RRID: AB_88384), and anti-mouse (Invitrogen, 62-6520, 1:5000, RRID: AB_88369).

### Flow cytometry

50,000 cells were seeded in 6-well plates. At the indicated times, cells were scraped and harvested into FACS tubes and centrifuged at 500 × *g* for 5 min. Then, supernatant was discarded, and cells were washed once with 1 mL PBS, and centrifuged again at 500 × *g* for 5 min. Then, supernatant was discarded again and 200μL of pre-heated Bodipy C11 581/591 at 4 μM in medium was added to the cells. Cells were incubated at 37°C for 30 min in the dark and gentle shaking of 150-200 rpm in a rocker was applied. Cells were centrifuged at 500 × *g* for 5 min and resuspended in 200 μL of PBS and analysed using flow cytometer (BD LSRFortessa™ Cell Analyzer). Firstly, cell debris (FSC-A vs SSC-A), doublets and aggregates (FSC-A vs FSC-H) were excluded. Then, 525_50 blue A intensity was used to measure ferroptosis.

### Cell proliferation and cell death measured by Incucyte®

Cells were seeded in 96-well plates (500 cells/well) and specific conditions were applied after 24 h of seeding. Incucyte® S3 Live-Cell Analysis System (Sartorius) was used to determine cell proliferation using a cell confluence mask. Specifically, four regions in the field of view were analysed per well at the specified time frequences using 10X objective. Data were captured in a phase channel for confluence, and in a green channel for cell death determination using SYTOX green fluorescent probe (ThermoFisher, S7020). Analysis was performed using the Incucyte® software, and readouts were % confluence and green fluorescent cells count, or total integrated green fluorescence intensity.

Image-iT® Lipid Peroxidation Kit (ThermoFisher, C10445) was used to measure lipid peroxidation in cells. Cumene hydroperoxide was used as a positive control to induce lipid peroxidation at a final concentration of 100 μM, and the cells were incubated for 1.5 h at 37°C with 5% CO_2_. Subsequently, BODIPY 581/591 C11 stain was added at a final concentration of 10 μM, and the plate was placed in the Incucyte®. Images were obtained at 10X magnification every hour for 11 h.

### Reactive oxygen species determination

To detect ROS production in real-time, cells were seeded in 24-well glass bottom plates and indicated conditions were applied at the specific times. 25 μM Menadione (Cambridge Bioscience, M079) was used as a positive control to induce ROS species. Intracellular ROS levels were measured using CellROX Deep Red Reagent (Invitrogen, C10422) at a final concentration of 5 μM. Imaging was performed using the Nikon Ti2 inverted microscope system (equipped with Okolab environment chamber and CO2 mixer) at 20X magnification every 20 minutes for 12 h. Images were taken at four different sites in each well, and the experiment was performed with two technical repeats. Image analysis was conducted using an image analysis pipeline developed in Cell Profiler 4.2.6 software. The GaussianFilter module was used to smooth the data before identifying primary objects. The IdentyPrimaryObjects module was then employed to identify the fluorescent area of the cell (cytoplasm). Objects touching the edges of the image were filtered out using the SplitOrMergeObjects and FilterObjects modules. The integrated intensity of the identified objects was calculated using the MeasureObjectIntensity module. Additionally, the area covered by the identified objects was calculated for data normalisation using the MeasureObjectSizeShape module. The raw data was then exported for further analysis.

### Cells irradiation in γ-irradiator

In order to irradiate cells cultivated *in vitro*, cells were seeded in different plate formats (500,000 cells in p6 wells, or 1,500,000 cells in 60 cm plates), and were subjected to ionising gamma (γ) radiation using a Caesium-137 irradiator (GSR D1) after 6h. Different radiation doses (3-15 Gy) were applied by timing exposure to the radioactive source, calculated according to current activity (measured within 1 year) and source distance. For control doses of 0 Gy plated cells were sham irradiated by placing in the Cs-137 irradiator for comparable times but without exposure to the radioactive source. Then, cells were reseeded for the subsequent experiment.

### Diet administration and cyst(e)inase injections

For cysteine/cystine free diet administration, mice were placed on cysteine/cystine free diet (Modified from Baker Amino Acid Diet 5CC7 TestDiet® Catalog num. 1819663-203) or control diet (Baker Amino Acid Diet 5CC7 TestDiet® Catalog num. 1812281) two weeks before tumour cell injection into the mammary fat pad, and were kept on the diet for the rest of the duration of tumour growth until 4 weeks after injection.

For cyst(e)inase administration, once metastases were detected in the lung of mice using microCT, 100 mg/kg of body weight of cyst(e)inase was administered intraperitoneally every other day until the endpoint of the experiment (around 2 weeks after initiation of the treatment).

### Microcomputed tomography (micro-CT)

To quantify the metastatic burden in the lung, a Quantum GX2 micro-CT imaging system (PerkinElmer) was used. Either control or tumour-bearing mice were anaesthetised using isoflurane and the CT scan was performed using the following parameters: acquisition FOV: 36 mm; reconstruction FOV: 25 mm; filter: Al 0.5mm + Cu 0.06mm; voltage: 90 kV; current CT: 88uA. Gating for respiratory monitoring was performed and scans were obtained from the expiration phase. Data analysis was performed using Analyze (Analyze Direct) software as described in (49).

### Mice radiation treatment in Xstrahl - Small Animal Radiation Research Platform (SARRP)

Mice were irradiated with 220 kV photon (X-ray) beams specifically in the lung region using the SARRP image guided radiotherapy according to the manufacture instructions. Briefly, mice were first anaesthetised using isoflurane and were placed inside the SARRP. A planning CT scan was obtained first using the Aluminium filter of the SARRP. Then, using the Muriplan software, X-rays were targeted to the lung tissue using a single isocentre with two collimated oblique opposing partial arcs at 180 degrees of distance, to avoid dose to nearby normal tissue such as the heart and contralateral lung. Total doses were set at 2, 3, 4 or 6 Gy according to the experiment, and the fields were shaped to the lung contour using a variable collimator and delivered using Copper filter to irradiate the mice.

### Statistical analysis

All experiments were performed at least in triplicate and repeated at least two or three times as indicated in the Figure legends. All data are represented as mean ± SD. As specified in figure legends, comparisons between two conditions were done using Student’s t test with *p* < 0.05, for comparisons between more than two conditions, one-way ANOVA was used for the “condition” factor (usually different cell lines), and Student-Newman-Keuls and Duncan test for multiple comparisons. Symbols,#,£,$,&,indicate the significance of α = 0.05. Furthermore, Shapiro-Wilk test was used to assess normal distribution of the experimental data and Dixon’s Q-test to identify outliers and Levene’s test to test homogeneity of variances. R software (version 4.5.1) was used to perform all statistical analyses. For scRNA sequencing or mRNA sequencing analysis, statistics are explained in each corresponding section.

## Results

### Glutathione levels are increased in mammary gland tumour-derived lung metastases compared to primary tumours

**Figure 1:**
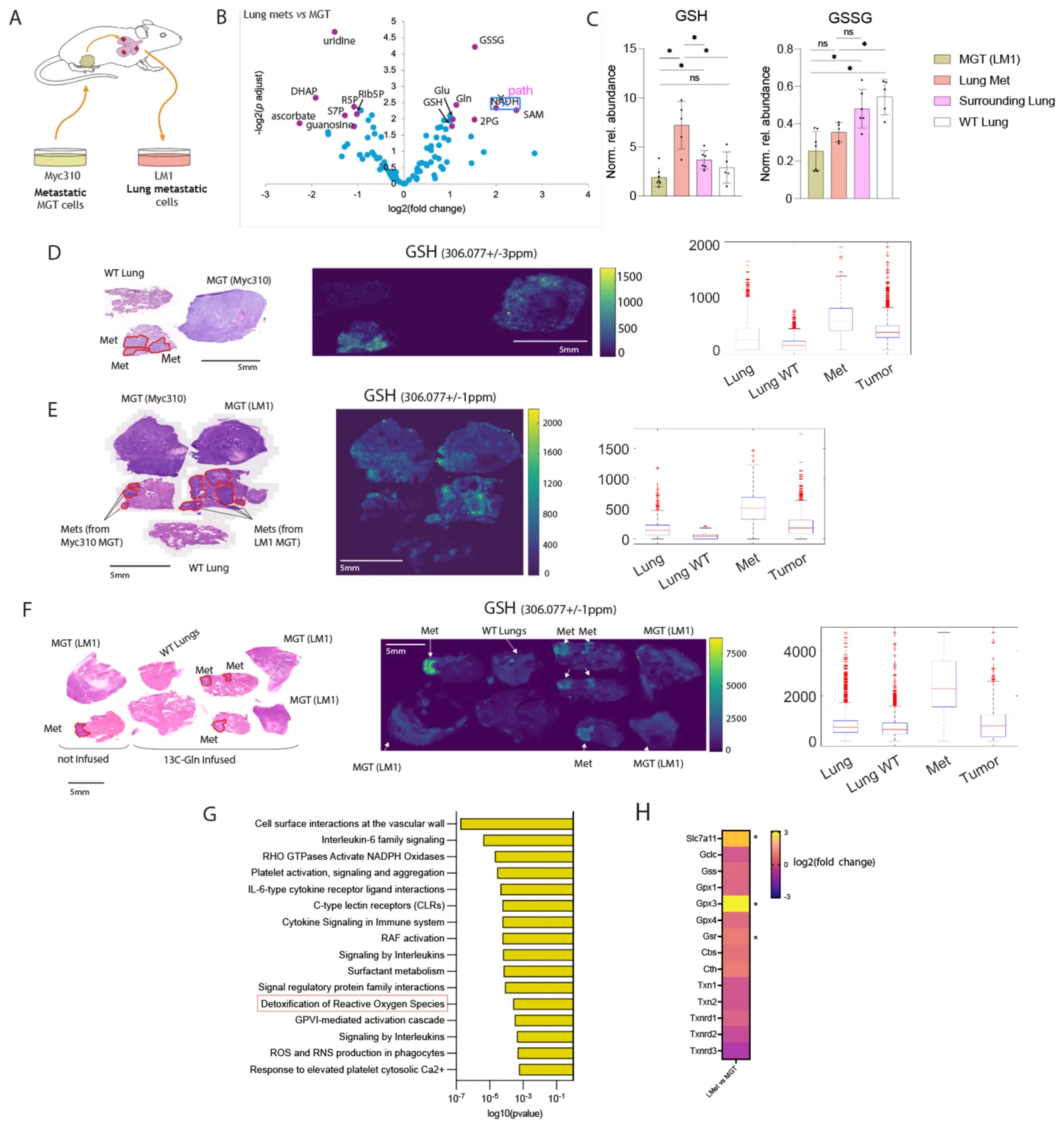
GSH levels and synthesis are increased in lung metastasis in comparison to MGTs. (**A**) Schematics showing the model of orthotopic mammary gland tumour (MGT) model used to compare lung metastasis and MGTs. (**B**) Volcano plot representing metabolite relative abundance with 2 fold change in lung metastasis vs MGTs. Purple dots are metabolites with >2 fold change and >0.05 *p* adjust value (*n =* 5 mice per group). (**C**) GSH and GSSG levels in MGTs, lung metastatic lesions, metastasis surrounding lung tissue, and normal lungs. Relative abundance is normalised to internal standard [U-^13^C,^15^N]valine and dry weight (*n* = 6-7 mice per group). Data are MEAN ± SD. * *p*<0.05 determined by *t* test. (**D**), (**E**), and (**F**) Single ion images of GSH detected by DESI-MSI in slices from MGT and lungs from Myc310-MGT-bearing mice and LM1-MGT-bearing mice. Consecutive H&E images and quantification of the ion intensities per pixel in the indicated regions are also shown. (**G**) Upregulated pseudobulk Reactome pathways in tumour cells from the metastatic lungs *vs* tumour cells from the MGTs in LM1-MGT-bearing mice as determined by single-cell RNA sequencing analysis. (**H**) Specific differentially expressed genes between metastatic lungs *vs* MGTs in LM1-MGT-bearing mice cell clusters in the same experiment as **G**. *n* = 3 mice per group. * - significantly upregulated pathways.

To determine the changes that occur during breast cancer cell metastasis to the lung, we used Myc310 cells, a cell line derived from one of the MMTV (mouse mammary tumour virus)-cMYC tumours. When injected orthotopically, Myc310 cells form mammary gland tumours (MGTs) that give rise to visible albeit small metastasis, which contrasts with mostly non-metastatic MMTV-cMYC MGTs. Since these lung metastatic lesions derived from orthotopic Myc310 MGTs are still too small for reliable analysis, we derived a new more metastatic cell line, LM1, from the lungs of Myc310 tumour-bearing mice (Fig. 1A). Injection of LM1 cells into the mammary fat pad of wild-type mice produced larger and more abundant lung metastases, which were easier to analyse.

Using untargeted analysis by liquid chromatography mass spectrometry (LC-MS) to compare LM1 metastases dissected from metastatic lungs and LM1 mammary gland tumours (MGTs), we found that the metastatic lesions presented increased levels of reduced glutathione (GSH), oxidised glutathione (GSSG), NADH, S-adenosylmethionine (SAM), glutamine, and glutamate, and decreased levels of metabolites from the pentose phosphate pathway (e.g. R5P, Rib5B) and nucleotide metabolism (e.g. uridine, guanosine) in comparison with MGTs (Fig. 1B). Targeted LC-MS analysis of the replicate set of samples further confirmed the increased levels of GSH in metastatic lesions in comparison with MGTs, lung tissue surrounding metastatic lesions as well as normal lungs (Fig. 1C). While in this set of samples there was a trend for increased GSSG levels in metastatic lesions in comparison with MGTs, the difference was not statistically significant (Fig. 1C). Of note, the levels of GSSG in both metastasis-surrounding lungs and normal lungs were significantly higher than in MGTs suggesting that the higher levels of GSSG in metastasis tissue samples can be a result of imperfect dissection. Consistent with the significantly higher levels of GSH in metastasis, GSH/GSSG ratio, which is indicative of the cell antioxidant capacity, was also higher in metastasis compared to all other tissues (Supplementary Fig. S1A). SAM and SAH levels in metastatic lesions of the second set were comparable to the levels in MGTs but were higher in lung tissues in comparison with MGTs (Supplementary Fig. S1B). Overall, targeted LC-MS results confirmed the increase in GSH and antioxidant capacity in metastasis compared to primary tumours.

To confirm the differences between metastases and primary tumours without dissecting metastatic lesions, we examined the metabolite distribution *in situ* by Desorption Electrospray Ionisation Mass Spectrometry Imaging (DESI-MSI)(18). DESI-MSI analysis supported our findings, as metastatic lesions had a significantly higher signal from an ion annotated as GSH in comparison with MGTs, surrounding lungs, as well as normal lungs (Fig. 1D, E, and F). DESI-MSI also revealed a high signal for glutamate, a precursor of GSH biosynthesis, in the metastatic lesions (Supplementary Fig. S1C and D). Furthermore, we confirmed glutamine catabolism to be a source of increased glutamate levels in metastatic lesions by identifying the distribution of ^13^C-glutamate in tissues from tumour bearing animals infused with [U-^13^C]glutamine (Supplementary Fig. S1C and D). However, we could not detect ^13^C carbons from glutamine in GSH within the time frame mice were infused (Data not shown).

Consistent with metabolomics and DESI-MSI analysis, single-cell RNA sequencing (scRNAseq) analysis of LM1 metastasis *vs* LM1 MGTs identified “Detoxification of Reactive Oxygen Species” among the upregulated processes, together with interactions with vascular wall, platelet activation, interleukin signalling, and Raf activation (Fig. 1G and Table S1). Specifically, the expression of genes encoding the cystine uptake transporter xCT, *Slc7a11*, and the GSH metabolism enzymes *Gpx3*, and *Gsr* was higher in metastasis compared to MGTs (Fig. 1H). Among the most downregulated processes, we found a general downregulation of translation and ribosomal RNA processing (Supplementary Fig. S1E and Supplementary Table S2), which can be a result of the oxidative stress(19,20). Taken together, these analyses demonstrated that, consistent with the previously published results(7), cMYC-driven mammary gland tumour lung metastases have upregulated glutathione metabolism in comparison to the primary tumours, supporting increased levels of GSH.

### Increased glutathione synthesis in lung metastases is fuelled by extracellular cystine rather than serine

**Figure 2:**
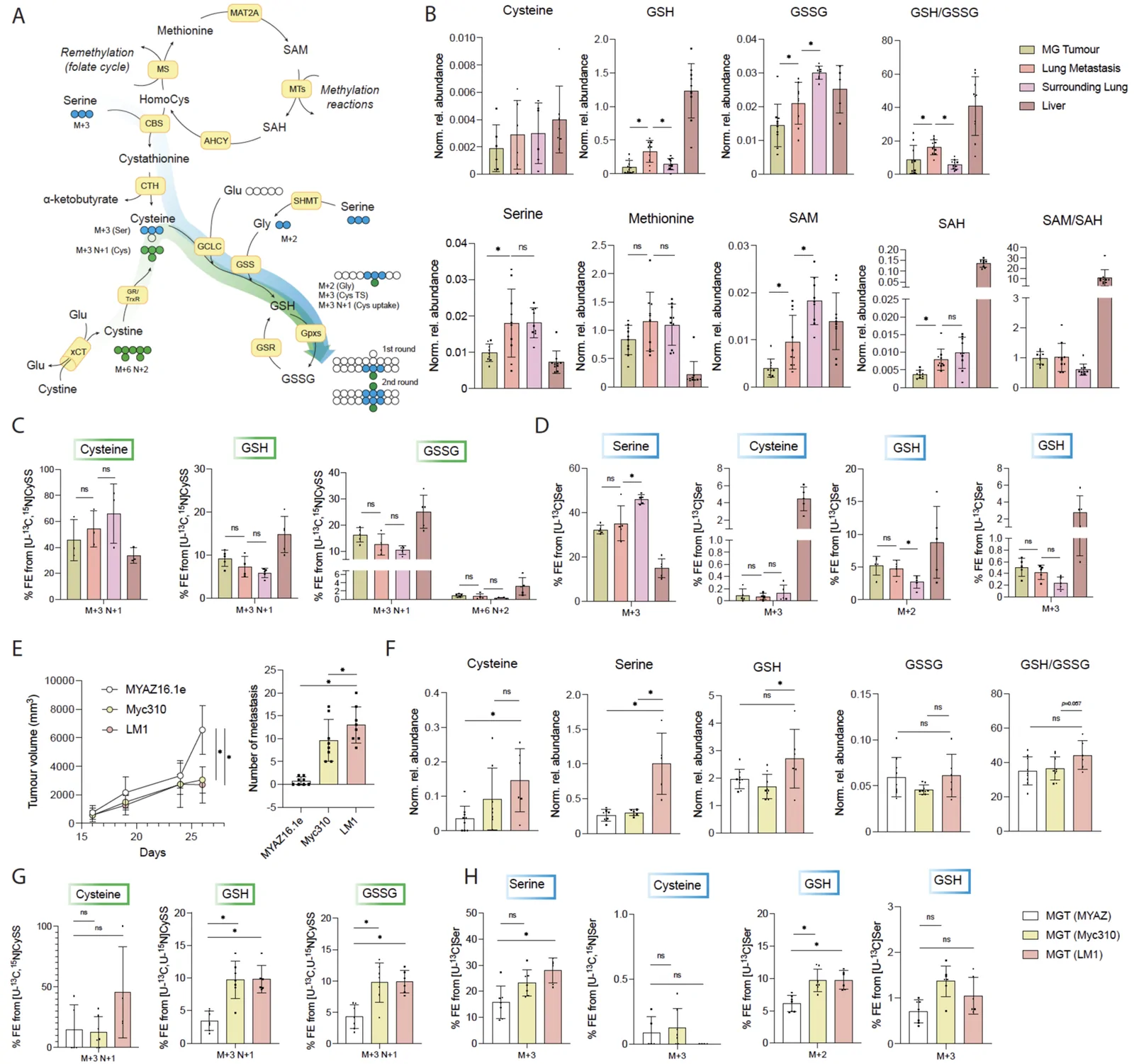
Extracellular cystine, rather than serine, fuels GSH synthesis in tumours and metastasis *in vivo*. (**A**) Schematics representing the stable isotope-resolved metabolomics strategy to resolve the source of cysteine used for glutathione synthesis. [U-^13^C,^15^N]cystine is used to track extracellular cystine uptake while [U-^13^C]serine is used to track *de novo* cysteine synthesis through the transsulfuration pathway. (**B**) – (**D**) Metabolite levels and their fractional enrichment in MGTs, lung metastasis, lung tissue surrounding the metastasis, and liver from LM1-MGT-bearing mice after 3 h of infusion of either [U-^13^C,^15^N]cystine in **C**, or [U-^13^C]serine in **D**. Relative abundance is normalised to internal standard [U-^13^C,^15^N]valine and dry weight (*n =* 5 mice per group for each stable isotope infusion). Data are MEAN ± SD. * *p*<0.05 determined by *t* test. Representative of three independent experiments. (**E**) Primary tumour growth and number of lung metastasis produced by MYAZ16.1e, Myc310 and LM1 cell-derived mammary fat pad tumours (*n =* 8 mice per group). Data are MEAN ± SD. * *p*<0.05 determined by *t* test. (**F**) – (**H**) Metabolite levels and fractional enrichments in MGT in **E**. Mice are infused with both [U-^13^C,^15^N]cystine (graph title highlighted in green) and [U-^13^C]serine (graph title highlighted in blue) (*n =* 8 mice per group). Data presented as MEAN ± SD, * p<0.05 determined by *t* test.

Cysteine is a key substrate for glutathione synthesis, which can either be produced from extracellular cystine or through *de novo* synthesis from serine through the TS pathway. Although various reports suggest that GSH synthesis in metastasis is increased, the source of cysteine fuelling GSH production has not been defined so far. To uncover the source of cysteine and glutathione in lung metastasis we used stable isotope-resolved metabolomics (SIRM). We infused mice carrying LM1 MGTs either with [U-^13^C,^15^N]cystine or [U-^13^C]serine. [U-^13^C,^15^N]cystine was used to determine cystine uptake and its contribution to glutathione synthesis while [U-^13^C]serine was used to determine serine contribution to glutathione synthesis and TS pathway activity (Fig. 2A). We used liver tissue as a positive control for the activity of both pathways as they are highly active in the liver (21). Consistent with our previous results, GSH levels were significantly increased in dissected lung metastases compared to the primary tumours and surrounding lungs (Fig. 2B). GSSG levels were also increased in both lung metastases and surrounding lungs compared to the primary tumours. The GSH/GSSG ratio was specifically increased in lung metastasis tissues (Fig. 2B). Stable isotope labelling revealed that the fractional enrichment (FE) of extracellular cystine atoms in GSH was markedly higher (~10% FE of M+3 N+1 GSH from [U-^13^C,^15^N]cystine) (Fig. 2C) than that from the TS pathway (~0.5% FE of M+3 GSH from [U-^13^C]serine) (Fig. 2D). The extent of the contribution from either cystine or serine to GSH pool was comparable between primary tumours and metastasis (Fig. 2C and D). Consistent with cystine being the primary source for cysteine and GSH synthesis in both MGTs and metastasis, cystine levels were found to be significantly decreased in the serum of tumour-bearing mice compared to wild-type mice (Supplementary Fig. S2A).

Since the contribution of cystine to GSH pool was similar between LM1 MGTs and derived lung metastasis, we wondered whether this is because the MGTs are formed from LM1 cells which are already metastatic. We therefore evaluated cysteine metabolism in MMTV-cMYC tumour-derived cell lines with different metastatic potential: MYAZ16.1e, which when orthotopically injected only produces micro-metastases after 4 weeks; Myc310, which gives rise to more metastatic lesions than MYAZ16.1e; and lung metastasis-derived LM1 with even higher metastatic potential (Fig. 2E and Supplementary Fig. S2B and C). To compare glutathione synthesis in these cell models *in vivo*, we injected the cells into the mammary fat pads of mice and used the same stable isotope tracing strategy as above to evaluate glutathione synthesis either from cystine or serine in both MGTs. Both Myc310 and LM1 MGTs had significantly higher cysteine than MYAZ16.1e MGTs, while serine and glutathione, were only significantly increased in LM1 MGTs (Fig. 2F). The levels and FE of cystine-labelled GSH and GSSG were significantly higher in MGTs derived from more metastatic Myc310 and LM1 cells compared to MGTs derived from less metastatic MYAZ16.1e cells (Fig. 2G).

Infusion of [U-^13^C]serine into tumour bearing animals resulted in increased serine labelled in three carbons (serine M+3) in LM1 in comparison with MYAZ16.1e MGTs, suggesting increased serine uptake (Fig 2A and 2H). Both Myc310 and LM1 MGTs also had higher FE of serine-derived GSH labelled in two carbons (GSH M+2), reflecting enhanced activity of the Glutathione synthase (GSS)-catalysed reaction. However, there were no significant changes in the incorporation of serine carbons coming from the TS pathway, represented by GSH M+3, between MGTs derived from the different cell lines (Fig. 2A, 2H and Supplementary Fig. S2D). Consistent with the metabolomics results, the expression of genes encoding the enzymes responsible for glutathione synthesis and turnover, *Gclc* and *Gsr*, was significantly higher in Myc310 and LM1 MGTs than in MYAZ16.1e MGTs, as determined by RT-qPCR. In contrast, the expression of Cystathionine β-synthetase (*Cbs*), the gene encoding the first and rate-limiting enzyme of the TS pathway, was significantly lower in Myc310 and LM1 MGTs than in MYAZ16.1e MGTs (Supplementary Fig. S2D).

Overall, these results demonstrate that increased metastatic potential of the mammary gland tumour cells is associated with increased synthesis of glutathione, primarily fuelled by cysteine derived from extracellular cystine. While metastatic cells also increase their usage of serine for glycine production, contributing to increased GSH synthesis, serine makes only a minor contribution to GSH synthesis through cysteine production by TS, regardless of the metastatic potential. The results also suggest that the increase in GSH synthesis from extracellular cystine happens early in the metastatic process, as more metastatic Myc310 cells derived from a primary MMTV-cMYC tumour already show increased contribution of cystine-derived carbons into GSH pool. Finally, comparison of LM1 lung metastases *vs* LM1 MGTs demonstrates that the further adjustment of metastatic cells growing in the lung environment is associated with a further increase in GSH synthesis and pools, but that the relative cystine contribution to the GSH pool stays the same.

### Cells with higher metastatic potential preserve increased glutathione synthesis from extracellular cystine in culture

**Figure 3:**
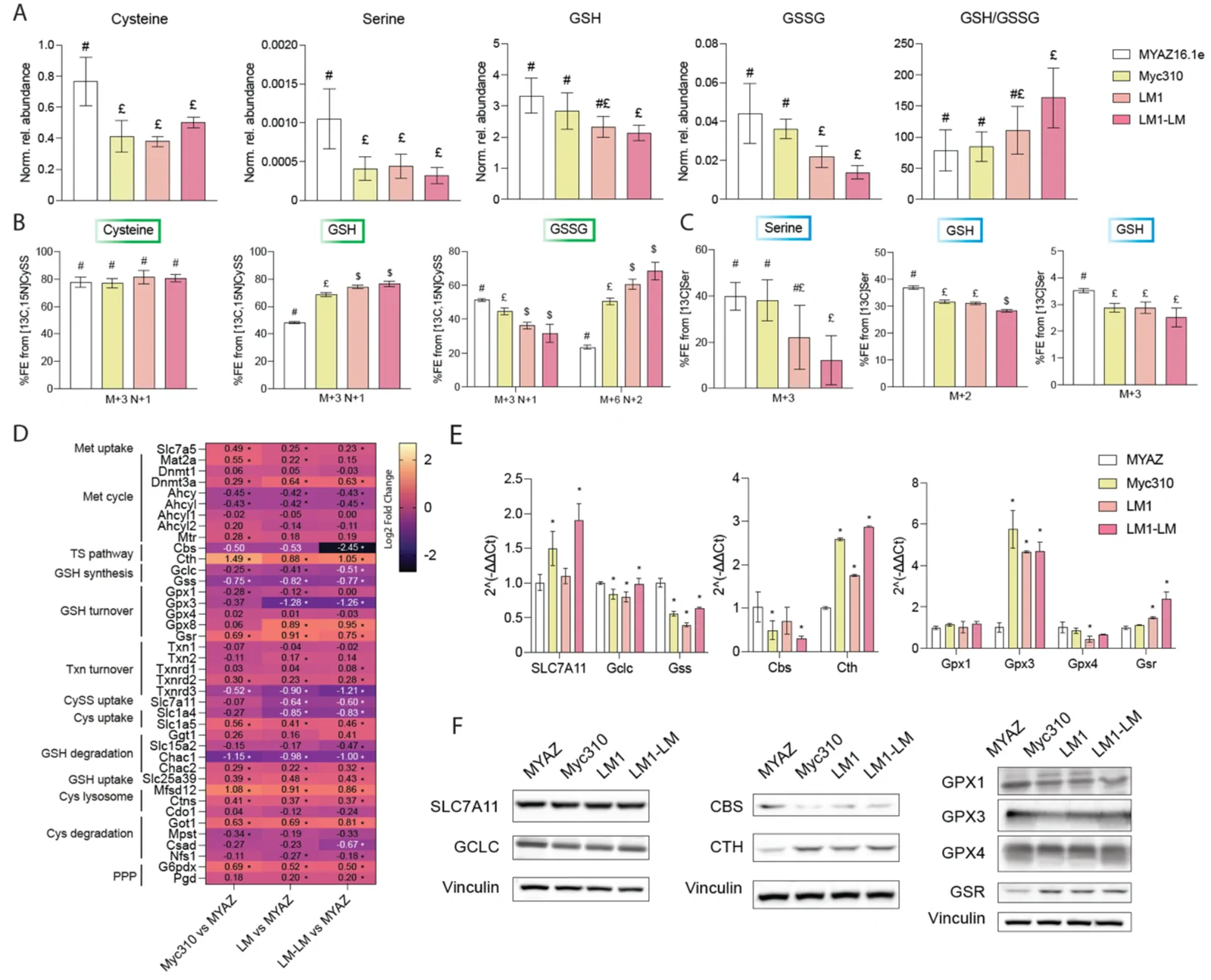
GSH synthesis is increased in highly metastatic cells compared to low metastatic cells. (**A**) – (**C**) Intracellular metabolite levels (in **A**) and fractional enrichments (in **B** and **C**) of cells incubated with [U-^13^C,^15^N]cystine for 6h in **B** or [U-^13^C]serine for 24h in **C** before metabolite extraction. Relative abundance of metabolites is normalised to internal standard [U-13C,15N]valine and protein concentration. Data are MEAN ± SD of technical replicates from three independent experiments with three replicates each. One-way ANOVA, SNK, and Duncan tests for multiple comparisons were performed for the factor cell line. Groups with different symbols (^#,£,$^) show significant differences with *α*=0.05. (**D**) Log2 fold changes of differentially expressed genes in Myc310, LMs (LM1, LM2, LM3) or LM-LMs (LM1-LM, LM2-LM, LM3-LM) cell lines compared to MYAZ cell lines (MYAZ16.1e, MYAZ12.3i, MYAZ6.6g). The experiment was performed using three biological replicates and three technical replicates. * *p* <0.05 (IHW adjustment). (**E**) mRNA levels of the indicated genes of Myc310, LM1, and LM1-LM cell lines relative to MYAZ16.1e cell line measured by RT-qPCR, with beta-actin expression used for normalization. Data are MEAN ± SD of technical replicates from three independent experiments with three replicates each. * *p* <0.05 determined by *t* test for Myc310, LM1 or LM1-LM compared to MYAZ16.1e. (**F**) Protein expression levels of the indicated proteins and cell lines measured by Western blotting. A representative of three independent experiments is shown.

To explore whether increased glutathione synthesis from cystine is a metabolic vulnerability of more metastatic cells, we used MYAZ16.1e, Myc310, LM1, and LM1-LM (a cell line derived from the metastasis of LM1 MGT-bearing mice) cell lines (Supplementary Fig. S2B). First, we evaluated whether the differences observed *in vivo* between tumours with low and high metastatic potential are preserved *in vitro*. Metabolomics analysis demonstrated that more metastatic cells had decreased levels of cysteine and serine in comparison with MYAZ16.1e cells, suggesting increased consumption of these glutathione precursors (Fig. 3A). However, the levels of GSH and GSSG were also decreased in metastatic cells, and the GSH/GSSG ratio was significantly higher in LM1 and LM1-LM cell lines in comparison with MYAZ16.1e cells (Fig. 3A). This could be indicative of a higher turnover of GSH-GSSG. Nevertheless, consistent with the *in vivo* results, incorporation of carbons from extracellular cystine into GSH after 6h of incubation with [U-^13^C,^15^N]cystine was significantly higher in more metastatic cells and increased with the metastatic potential (Fig. 3B). GSSG M+6 N+2 FE, which flags the incorporation of two equivalents of cysteine to the GSH system, indicating two cycles of GSH-GSSG turnover (Fig. 2A), also gradually increased in more metastatic cells compared to less metastatic cells (Fig. 3B, Fig. 2A), suggesting a higher glutathione turnover rate. To evaluate the activity of TS in this cell systems, we incubated cells with [U-^13^C]serine for 24 h to account for the significantly lower contribution of serine-derived carbons to the GSH pool. In agreement with the *in vivo* results, a significant decrease in the FE of the serine-derived GSH pool was observed in more metastatic vs less metastatic cells (Fig. 3C).

To evaluate if metabolic changes during the metastatic process are driven by the activity of any specific transcription factors or genetic drivers, we performed bulk mRNA sequencing of the same cell lines and biological replicates followed by Gene Set Enrichment Analysis (GSEA) and Transcription Factor Targets prediction analysis. Although we were not able to identify any transcription factor signatures (Supplementary Fig. S3A and B), the expression of genes encoding enzymes of glutathione metabolism was consistent with metabolic changes observed both *in vivo* and *in vitro:* decreased activity of the TS pathway in more metastatic cells revealed by SIRM was associated with decreased levels of mRNA and protein for CBS, the rate-limiting enzyme of the TS pathway (Fig. 3D, 3E, 3F, and Supplementary Figure S3C) while the expression of Cystathione γ-lyase (CTH), an enzyme downstream of CBS, was increased; GSR, which recycles GSSG back to GSH, was increased in metastatic cell lines at both mRNA and protein levels, consistent with the higher GSH-GSSG turnover determined by SIRM (Fig. 3E, 3F, and Supplementary Figure S3C). Finally, we observed a drastic increase in *Gpx3* gene expression in more metastatic cell lines. However, this increase was not recapitulated at the level of protein expression (Fig. 3E, F, and Supplementary Figure S3C), potentially because GPX3 is normally exported outside of the cell for the protection of the extracellular matrix and fluids(22). Taken together, our integrated metabolomics and transcriptomics analyses strongly suggest that as cells acquire greater metastatic potential, they undergo a metabolic reprogramming involving enhanced GSH synthesis and reduced activity of the TS pathway.

### Cysteine and cystine are indispensable for cells with high metastatic potential

**Figure 4:**
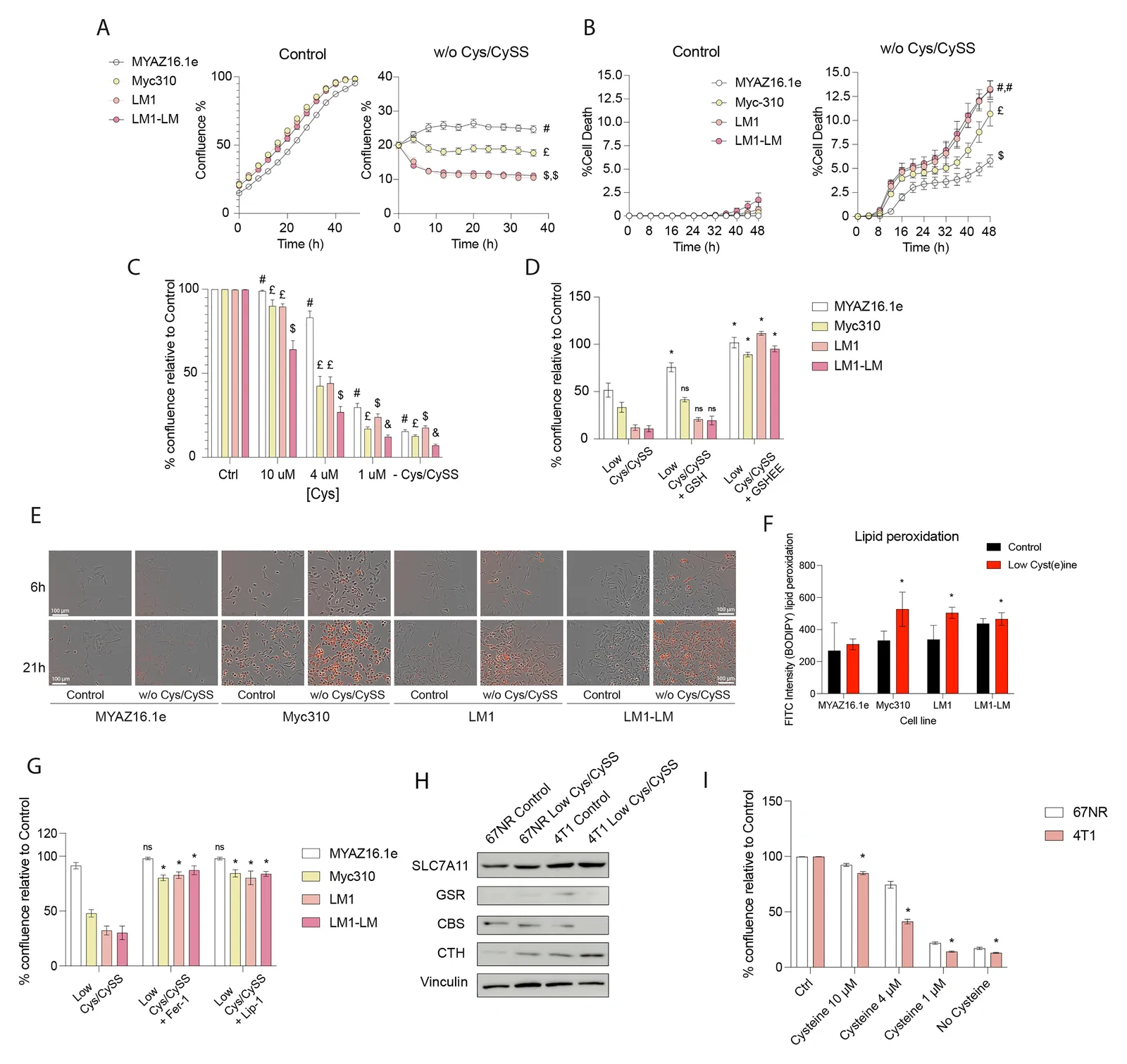
Deprivation of both cysteine and cystine decreases proliferation and induces higher ROS levels, lipid peroxidation, and cell death in metastatic cells. (**A**) The confluence of cells grown either in the presence or absence of cysteine/cystine measured by Incucyte®. (**B**) Cell death measured by Incucyte® using SYTOX green dye measured in the presence or absence of cysteine/cystine. (**C**). Cell confluence measured by Incucyte® at different concentrations of cysteine/cystine relative to the complete media at 72h. (**D**) The confluence of cells grown under low cysteine/cystine conditions (4 μM of each) in either absence or presence of either 0.5 mM GSH, or 0.5 mM ethyl ester glutathione (GSHEE) measured by Incucyte® at 72h. (**E**) CellROX Deep Red staining of cells with or without cysteine/cystine, images taken by Incucyte®. (**F**) Lipid peroxidation measured by BODIPY dye 6h after cysteine/cystine deprivation analysed by flow cytometry. FITC (510 emission) is represented as indicative of lipid peroxidation. * *p*<0.05 determined by *t* test of Low Cys/CySS vs Control. (**G**) Cell confluence measured by Incucyte® under low cysteine/cystine conditions (4 μM of each) either in the presence of absence of either 2 μM ferrostatin or 0.1 μM liproxtatin at 72h. (**H**) Protein expression levels of the indicated proteins in 4T1 and 67NR grown in either normal or low cysteine/cystine measured by Western blotting. (**I**) Cell confluence measured by Incucyte® at different concentrations of cysteine/cystine relative to the complete media at 72h. In all cases, a representative of three independent experiments with three technical replicates each is shown; Data are MEAN ± SD of technical replicates. One-way ANOVA, SNK, and Duncan tests for multiple comparisons were performed for the factor cell line. Groups with different symbols (^#,£,$,&^) show significant differences with *α*=0.05; and * *p*<0.05 determined by *t* test.

To investigate whether the increased production of glutathione from cystine could be exploited as a vulnerability of more metastatic cells, we evaluated the sensitivity of these cells to the deprivation of various amino acids that contribute to glutathione synthesis (Supplementary Fig. S4A). Only the absence of both cystine and cysteine affected the proliferation and viability of Myc310, LM1 and LM1-LM cells to a greater extent than MYAZ16.1e cells (Fig. 4A, 4B, and Supplementary Fig. S4A and B). In addition, reducing cystine and cysteine levels consistently caused a decrease in cell confluency across various concentrations, with the extent of this reduction inversely proportional to the metastatic potential of the cell lines (Fig. 4C). Tumouroid-type cultures, which better mimic real tumour conditions, confirmed that the more metastatic cell lines were more sensitive to cysteine/cystine depletion in this type of culture (Supplementary Fig. S4C). Furthermore, the inhibitor of cystine uptake by xCT transporter, imidazole ketone erastin, had lower IC50 values in more metastatic cells than in MYAZ16.1e, confirming the higher dependence of metastatic cells on cysteine/cystine availability (Supplementary Fig. S4D). To determine whether GSH was a key product of cysteine metabolism to sustain cell proliferation and survival, we incubated cells with GSHEE, a permeable form of GSH, which completely rescued the confluency of metastatic cells under low cysteine/cystine levels (Fig. 4D).

Cysteine/cystine deprivation increases reactive oxygen species (ROS) and induces tumour cell death by ferroptosis(23). Indeed, we observed that upon cysteine/cystine deprivation, both ROS levels and lipid peroxidation were significantly higher in Myc310, LM1, and LM1-LM cells than in MYAZ16.1e cells (Fig. 4E and 4F, and Supplementary Fig. S4E). Consistently, the ferroptosis inhibitors ferrostatin and liproxtatin were able to rescue the confluence of metastatic cells in the presence of low cysteine/cystine levels (Fig. 4G). Therefore, cell lines with higher metastatic potential are more sensitive to cysteine/cystine deprivation, which triggers ferroptosis-mediated cell death, making them addicted to the presence of the amino acid cysteine.

Finally, we determined whether the same metabolic phenotype of cysteine addiction in the cells with high metastatic potential was observed in another model of murine lung metastasis. We chose the mouse isogenic cell lines 67NR and 4T1, representing a non-metastatic and metastatic triple negative breast tumour cells, respectively (24). Consistent with their higher metastatic potential, 4T1 cells, which had higher protein levels of SLC7A11 and GSR (Fig. 4H, and Supplementary Fig. S4F), were more sensitive to low cysteine/cystine levels compared to the non-metastatic 67NR model (Fig. 4I). Similar to MMTV-cMYC-derived cell lines with higher metastatic potential, the proliferation of 4Τ1 cells under low cysteine/cystine conditions was rescued by ferroptotic inhibitors, suggesting a similar mechanism of cell death by ferroptosis (Supplementary Fig. S4G).

### Downregulation of the transsulfuration pathway underlies the dependence of more metastatic cells on cysteine/cystine availability

**Figure 5:**
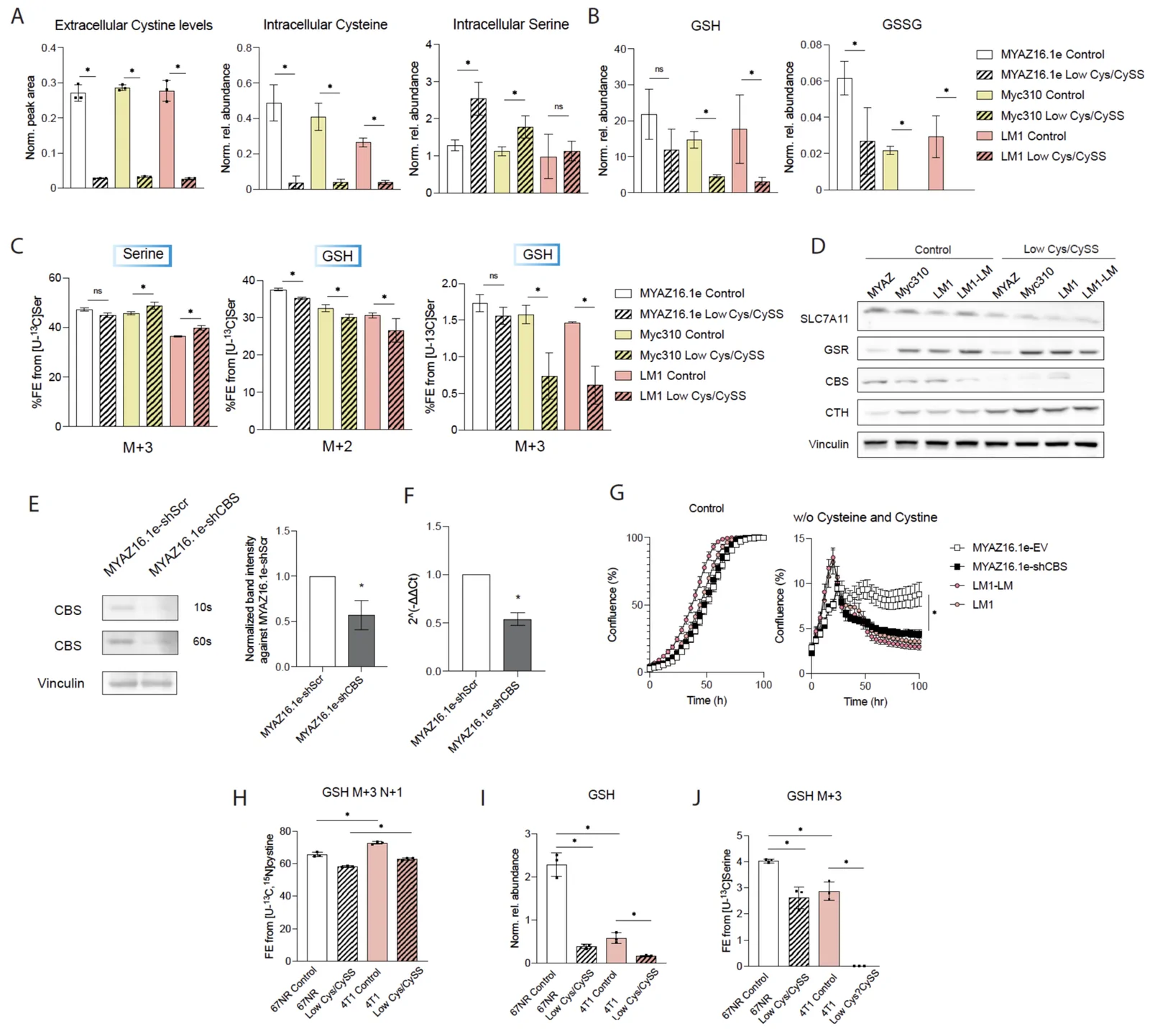
High metastatic cells have a decreased transsulfuration pathway. (**A**) - (**C**) Total abundance (in **A** and **B**) and fractional enrichments (in **C**) of metabolites in indicated cells incubated with [U-^13^C]serine for 24h under either control or under low cysteine/cystine conditions (4 μM of each). Abundance was normalised to internal standard [U-13C,15N]valine and protein concentration. (**D**) Protein expression levels of the indicated proteins and cell lines measured by Western blotting. (**E**) CBS protein expression level in the indicated cell lines. Percentage of protein expression is measured using ImageJ. (**F**) mRNA levels of Cbs gene measured by RT-qPCR, with beta-actin expression used for normalization. (**G**) The confluence of the indicated cell lines grown either with or without cysteine/cystine measured by Incucyte®. (**H**) – (**J**) Total abundance (in **I**) and fractional enrichments (in **H** and **J**) of metabolites in 67NR and 4T1 cells incubated with either [U-^13^C,^15^N]cystine for 6h or [U-^13^C]serine for 24h under low cysteine/cystine conditions (4 μM of each). In all cases, a representative of three independent experiments is shown. Data are MEAN ± SD of technical replicates. * *p* < 0.05 determined by *t* test.

To further explore why more metastatic cells are more sensitive to cysteine/cystine deprivation, we used metabolomics analysis. As expected, culturing cells in low cysteine/cystine conditions resulted in significantly decreased levels of both extracellular cystine and intracellular cysteine, while intracellular levels of serine were increased at both 24h and 52h (Fig. 5A and Supplementary Fig. S5A). Notably, under low cysteine/cystine conditions at both 24h and 52h, GSH levels were only significantly decreased and GSSG levels were undetectable in more metastatic cell lines, Myc310 and LM1 (Fig. 5B and Supplementary Fig. S5B).

To evaluate whether the activity of the TS pathway was affected by low cysteine/cystine conditions, we cultured cells with [U-^13^C]serine. While MYAZ16.1e cells maintained the activity of the TS at 24h at low cysteine/cystine levels, as indicated by GSH M+3 FE, the TS pathway-derived GSH carbons significantly decreased in Myc310 and LM1 metastatic cells (Fig. 5C). In contrast, FE of GSH M+2, which is produced from serine-derived glycine, was decreased to the same extent in all cell lines. The difference in TS activity between more metastatic cell lines and MYAZ16.1e was still observed at 52h of cysteine/cystine reduction (Supplementary Figure S5C). Consistent with stable isotope labelling analysis, the protein levels of CBS, decreased in cells with high metastatic potential, were further decreased upon cysteine/cystine reduction (Fig. 5D and Supplementary Figure S5F). While the levels of SLC7A11 (xCT) protein were slightly decreased in response to cysteine/cystine reduction, the levels of CTH increased (Fig. 5D and Supplementary Figure S5F). Consistently RNAseq analysis of cells cultured in the absence of cysteine/cystine but for a short period of time (6 hours), demonstrated upregulation of *Cth* in all cell lines (Supplementary Figure S5D and S5E). Furthermore, *Cha*c1, a gene encoding a GSH-degrading enzyme, was strongly upregulated at the transcriptional level in all cell lines (Fig S5D), suggesting a compensatory upregulation of GSH degradation, which may help cells to recover cysteine levels. GSEA of this dataset showed upregulation of pathways that respond to amino acid deprivation, and heme deficiency in all cell lines in responce to cysteine/cystine deprivation (Fig S5E).

Overall, our results demonstrated that TS pathway, which is lower in more metastatic compared to less metastatic cells (Fig. 3C), is further decreased in more metastatic cells in cysteine/cystine-restricted conditions. We hypothesised that this suppression of TS activity may be a reason for the inability of metastatic cells to sustain GSH levels under these conditions. To confirm that the reduced activity of CBS and consequently of the TS pathway are a cause of the dependency of more metastatic cells on extracellular cysteine/cystine availability, we knocked down the *Cbs* gene in MYAZ16.1e cells. Decreasing CBS expression (Fig. 5E and 5F) was sufficient to make MYAZ16.1e dependent on cysteine/cystine availability, both in 2D and tumoroid-type cultures (Fig. 5G, and Fig. S5G), mimicking the effect of cysteine/cystine deprivation in cells with high metastatic potential. These data and the results above demonstrate that a higher requirement for GSH synthesis and impairment of the TS pathway make metastatic cells more dependent on cysteine/cystine availability.

To confirm whether the relationship between cysteine/cystine deprivation and TS activity also exists in 67NR/4T1 cell model, we compared cystine and serine contribution to GSH pools in these cells. Consistent with the results from MMTV-MYC derived cell lines (Fig. 3B), metastatic 4T1 cells had significantly higher FE of cystine-derived GSH M+3 N+1 in both control and low cysteine/cystine conditions (Fig. 5H) despite having a lower glutathione pool (Fig. 5I). The contribution of serine to GSH through the TS pathway was lower in 4T1 in comparison to 67NR cells in control conditions (Fig. 5J); and while under low cysteine/cystine conditions it only decreased by 30% for non-metastatic 67NR cells, serine incorporation in GSH was undetectable in metastatic 4T1 cells (Fig. 5J). Consistent with these results and results from MMTV-MYC derived cell lines, CBS protein levels decreased while CTH levels increased only in 4T1 cells under low cysteine/cystine conditions (Fig. 4H and Supplementary Fig. S4F). Overall, these results further confirm that the TS pathway is downregulated in more metastatic cells compared to less metastatic cells.

### Combining cyst(e)inase with radiation successfully reduces lung metastases

**Figure 6:**
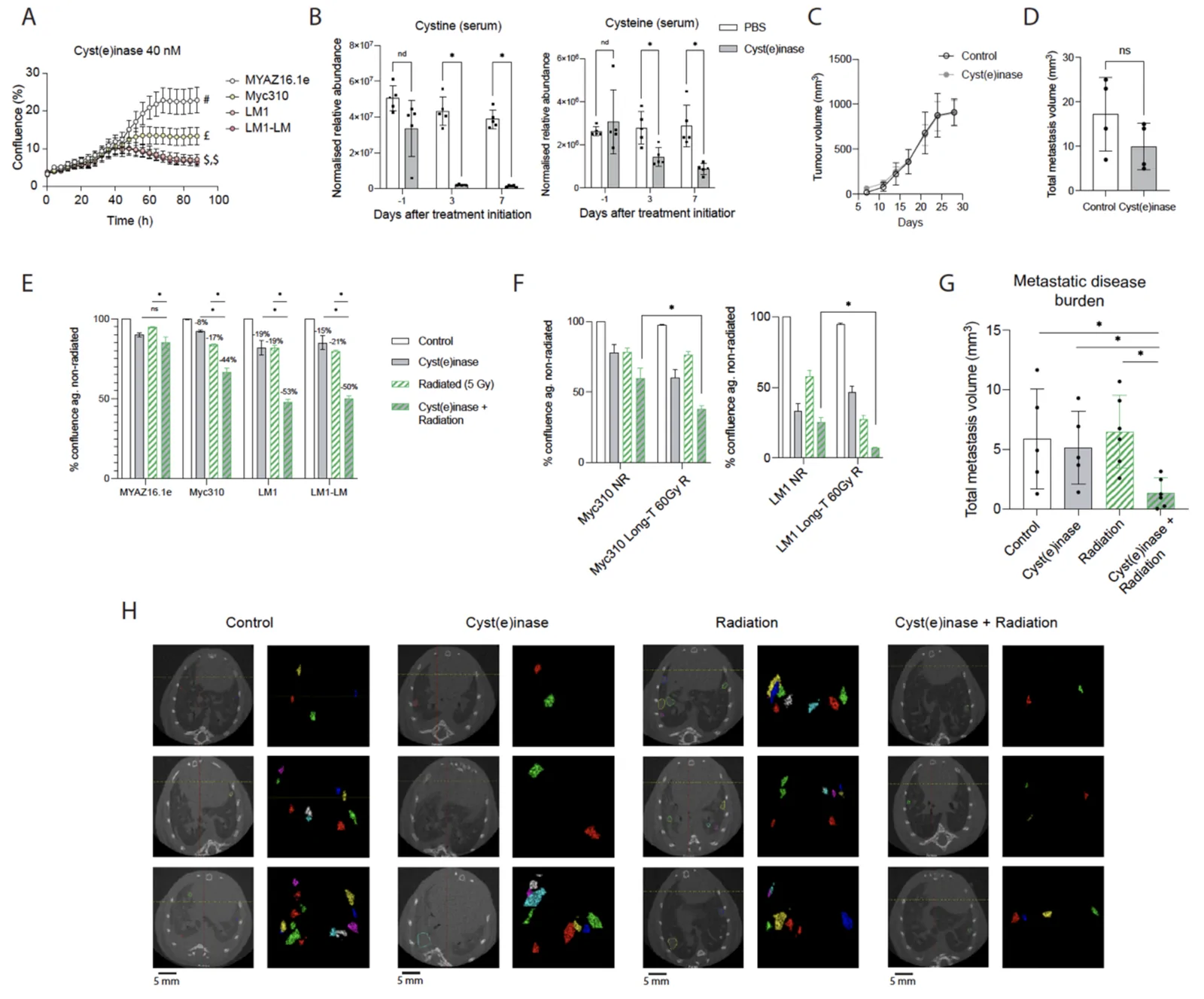
A combination of Cyst(e)inase and radiation reduces proliferation of metastatic cells *in vitro* and growth of lung metastasis *in vivo*. (**A**) The confluence of cells treated with 40nM cyst(e)inase as measured by Incucyte®. A representative of three independent experiments with three replicates each is shown. Data are MEAN ± SD of technical replicates. One-way ANOVA, SNK, and Duncan tests for multiple comparisons were performed for the factor cell line. Groups with different symbols (^#,£,$^) show significant differences with *α*=0.05. (**B**) Metabolite abundance in the serum of FBV/NJ mice with the orthotopic injection of LM1 cells treated with 100 mg/kg of cyst(e)inase for 7 days (every other day) starting 3 weeks after tumour cell injection. (*n* = 4-5 mice per group). Data are MEAN ± SD. * *p*<0.05 determined by *t* test. (**C**) The growth of tumours in **B**. Data are MEAN ± SD. (**D**) Total metastasis volume calculated from the images in Supplementary Fig. S6F from the experiment in **B** using AnalyzeDirect software. (**E**) The confluence of cells treated with either 20nM Cyst(e)inase, or 5Gy irradiation, or both measured by Incucyte® at 72h. The data are presented as confluence relative to the non-radiated control of every cell line. A representative of three independent experiments with three replicates each is shown. Data are MEAN ± SD of three technical replicates. * *p*<0.05 determined by *t* test. (**F**) Cell confluence at 72h measured by Incucyte® of cells cultured in DMEM:F12 and treated with either Cyst(e)inase 20 nM, radiated (5Gy) using a Cesium radiator, or both treatments. Confluence relative to non-radiated control of every cell line, that was either “NR” (non-radiated) or “Long-T 60Gy R” (long-term radiated twice every week for 4 weeks, a total of 60Gy dose). A representative of three independent experiments with three replicates each is shown. Data are MEAN ± SD of three technical replicates. * *p*<0.05 determined by *t* test. (**G**) Total metastatic volume in FBV/NJ mice with the orthotopic injection of LM1 cells treated with either 100 mg/kg of Cyst(e)inase or lung-targeted 4Gy irradiation, or both treatments as described on Material and Methods. (*n* = 5-6 mice per group). Data are MEAN ± SD. A representative of two independent experiments is shown. The other experiment is shown in Figure S6K. * *p*<0.05 determined by *t* test. (**H**) Representative microCT images of lungs from the experiment in **G**.

The high sensitivity of more metastatic cells to cysteine/cystine deprivation prompted us to evaluate the effect of a cysteine-free diet (CFD) on tumour growth and metastasis formation after injection of LM1 cells into the mammary fat pad of wild-type mice. The diet did not affect mouse body weight, and the levels of cysteine and cystine remained unchanged in the blood of treated animals (Supplementary Fig. S6A - C), likely due to the compensatory cysteine synthesis in the liver (25). Consistently, no differences were observed in the tumour volume or their metastatic burden (Supplementary Fig. S6D and E). Therefore, as an alternative and more effective method for reducing levels of cysteine in the blood, we used cyst(e)inase, a synthetic enzyme designed to specifically degrade free cysteine and cystine (17). As expected, treatment with cyst(e)inase mimicked the effect of cysteine/cystine withdrawal in the set of MMTV-cMYC derived cell lines *in vitro* (Fig. 6A). Furthermore, cyst(e)inase treatment in LM1 MGT-bearing mice for 10 days effectively reduced blood levels of both cystine and cysteine (Fig. 6B), with the latter one to a lesser extent. However, Consistent with the fast intracellular conversion of cystine into cysteine, we did not detect cystine either in lungs or metastatic lesions (data not shown), cysteine levels were significantly reduced by cyst(e)inase treatment in the metastasis-surrounding lungs but not in metastatic lesions (Supplementary Fig. S6G). Consistently, cyst(e)inase treatment did not significantly reduce tumour volume or metastatic burden as measured by micro-computed tomography (microCT) (Fig. 6C and D, and Supplementary Fig. S6F). These results suggested that during cyst(e)inase treatment metastatic tumours manage to maintain their cysteine levels by perhaps taking up the residual cysteine and cystine from the surrounding tissues and circulation. Given the established role of radiotherapy in the treatment of metastatic disease, including lung metastasis(26), and the fact that ionising radiation generates ROS, we hypothesised that further increasing the levels of ROS in the metastases would increase their demand in GSH pools and make them even more dependent on maintaining GSH homeostasis, and hence on cysteine/cystine availability. Indeed, combining radiotherapy with cyst(e)inase treatment *in vitro* efficiently reduced the survival of metastatic cells (Fig. 6E). A prolonged period of treatment with fractionated radiation (up to a total cumulative dose of 60Gy) further increased the synergy between radiation and cyst(e)inase in Myc310 and LM1 cells (Fig. 6F).

To evaluate the effect of the combination of cyst(e)inase and radiation *in vivo*, we first optimised the dose of focal radiotherapy to metastatic mouse lung that would achieve a significant but incomplete reduction of metastatic lesions in LM1 MGT-bearing mice (Supplementary Fig. S6H) without affecting their body weight (Supplementary Fig. S6I). Consistent with the *in vitro* results, the combination of cyst(e)inase treatment with radiation targeted to the lungs produced a synergistic reduction in metastatic burden compared to either monotherapy or control, while having no significant effect on primary mammary tumour growth (Fig. 6G and H, Supplementary Fig. S6J, S6K, and Supplementary Fig. S7). Crucially, although serum levels of cystine and cysteine were still decreased (Supplementary Fig. S6L and M), the combination treatment was well tolerated, without any observed body weight loss or other adverse effects (Supplementary Fig. S6N). Therefore, these results confirm that the dependency of metastatic cells on cysteine/cystine can be therapeutically exploited, particularly when combined with ROS-inducing therapies such as radiotherapy.

## Discussion

Uncovering mechanisms underlying metastatic process and identifying novel ways to either prevent or target metastatic disease is one of the most urgent needs in oncology research. Both intrinsic and extrinsic tumour cell metabolic activities have been shown to play a role in the establishment and growth of metastasis. In recent years, the antioxidant capacity of tumour cells has been identified to be essential for successful metastasis(5,27,28). Here, we confirmed using different metabolomics techniques that GSH levels are indeed upregulated in a model of mouse mammary gland tumour-derived lung metastasis induced by cMYC. We furthermore revealed extracellular cystine as the main source of GSH and shown that cells with high metastatic potential depend on the extracellular cystine to survive, possibly due to downregulated *de novo* cysteine synthesis. Finally, considering this vulnerability, we propose that combining cyst(e)inase with radiotherapy can be an effective approach to limit metastatic spread in breast cancer.

Efforts to uncover the metabolic adaptations of metastasis have been hindered by the challenges of reliably analysing metabolism in metastatic lesions. Conventional approaches often rely on time-consuming and inaccurate tissue dissection, which can compromise tissue integrity and conceal the specific cell types the metabolic phenotypes belong to(29). In most of the cases the conclusions are made based on either RNA sequencing or bulk tissue metabolomics results. However, the DESI-MSI approach we used in this study was a promising alternative strategy for spatially validating the upregulation of GSH specifically within lung metastases. Another method to distinctively uncover the changes in metastatic cells from other cells in the metastatic microenvironment, is scRNA sequencing. This technique revealed the upregulation of antioxidant pathways during the metastatic process. In addition, pathways related to interactions with the vascular wall, immune cells, and platelets were significantly upregulated in metastatic cells in comparison with primary mammary gland tumours, likely reflecting the adaptations associated with the cell journey through the circulation system from the mammary fat pad to the lung. Indeed, the acquisition of GSH/GSSG molecules from platelet mitochondria has been described as a key process for osteosarcoma lung metastasis by Zhang et al (30). We also found Raf activation as one of the significantly increased pathways, indicating a possible upregulation of the MAPK signalling pathways. Finally, we found translation and rRNA processing to be downregulated in metastatic cells, which could be explained by oxidative stress inhibiting rRNA processing (19,20).

The main source of increased GSH synthesis in metastatic cells remains undefined. Depending on the tissue of origin different cancer types have different source preferences(9). Here we demonstrate that the GSH synthesis from cystine-derived cysteine is significantly increased in tumours derived from metastatic cells in comparison with non-metastatic tumours, driven by increased expression of GCLC and increased fractional contribution of cystine to the glutathione pool, suggesting that this reprogramming occurs during the acquisition of metastatic traits. Comparison between the primary and metastatic sites revealed a further increase in the glutathione pool and GSH/GSSG ratio. These results suggest that the significantly increased activity of GSH synthesis from cystine allows cancer cells to survive during extravasation and circulation. Another boost in GSH synthesis, possibly stimulated by the lung environment, may also lead to increased GSH levels and would support metastatic growth in the lung.

Yoon et al (9) recently concluded that TS activity is generally low in cancer. Here, we show for the first time that like primary tumour cells, metastatic cancer cells mainly rely on importing extracellular cystine to make glutathione, rather than synthesising *de novo* cysteine from serine via the TS pathway. Importantly, the contribution of TS pathway to the glutathione pool is further decreased in metastatic cells in comparison with non-metastatic cells. The preference of tumour cells for using cystine for GSH synthesis can be due to relatively slow speed of TS pathway and its reliance of homocysteine availability (31,32). TS pathway withdraws a molecule of homocysteine from the methionine cycle, a crucial pathway for maintaining cellular methylation patterns. Decreasing TS activity, thus, can be essential for the regulation of these processes during metastasis. While both CBS and CTH expression are known to be upregulated in response to oxidative stress and cysteine deprivation (10), our results unexpectedly revealed the increase of CTH expression but decrease of CBS expression in more metastatic cells and further in response to cystine/cysteine deprivation. These results may indicate the differential regulation and role of these two enzymes: CBS withdraws carbons from the methionine cycle and thus may compete with methylation-dependent processes. In contrast, CTH captures cystathionine coming from both intracellular and extracellular sources and, since its activity does not directly affect methionine cycle, it may be required for the maximised activity of TS when CBS activity is supressed. However, the mechanisms of TS enzyme regulation during metastatic process would require further investigation.

Consistent with our previous results and the results of others, our current results demonstrate cysteine/cystine dependency of the metastatic cells (11,12,33,34). Our results indicate that this sensitivity may be determined by both high demand for GSH to compensate for the higher levels of ROS production and the inefficiency of the TS pathway. To try to target cysteine addiction of metastatic cells *in vivo*, we first tried a cysteine/cystine-free diet as it was previously reported to successfully reduce glioma progression(35). In our murine model, the cysteine/cystine-free diet did not deplete cysteine/cystine levels in the serum of mice and did not have any effect on metastasis progression. We therefore considered the use of an inhibitor of either cystine uptake (36) or glutathione synthesis(37). However, these inhibitors have proven challenging to translate into clinical practice due to the lack of specificity, poor solubility, and liver toxicity in animal models, as they deplete the glutathione pool, crucial for the detoxification capacity of the liver (38,39). To overcome these limitations, we used a novel cysteine/cystine degrader, cyst(e)inase(17), which has proven effective in prostate and breast primary tumour mouse models and is well tolerated in animal models (40,41). Importantly, cysteine/cystine depletion in the blood would affect metastatic cells due to their addiction to cysteine/cystine availability, while other organs such as the liver, or the brain, will still be able to produce cysteine/cystine through TS pathway activity. In our model of mammary gland tumour-derived lung metastasis, cyst(e)inase successfully reduced cysteine/cystine levels in the serum but was insufficient to reduce metastatic growth. This could be due to a limited capacity for degrading the monomeric cysteine, as described before(17). A new degrader with the ability to degrade both reduced and oxidised forms would be of use.

To further boost the reliance of metastatic cells on GSH and hence dependence on cysteine/cystine availability, we used focal radiation to enhance ROS levels specifically in the metastatic lesions. Combining cyst(e)inase with directed radiotherapy of the lung synergistically and significantly reduced the metastatic burden. Importantly, the combination treatment was well tolerated by the animals. The same combination was used in a primary melanoma tumour model to prove that both radiation and immunotherapy-derived cystine transporter inhibition have ferroptosis as a common mechanism (42). Here, we propose this combination as a potential treatment to tackle metastatic spread of breast cancer to the lung. Finally, although in our present work we focused on breast cancer metastatic models, we propose that combining radiation or any other ROS inducing agents with cyst(e)inase may be an effective and less toxic therapeutic strategy in other types of metastatic disease.

## Supplementary Material

Supplementary Fig. S1

## Data Availability

Data from bulk RNA sequencing of cell lines can be found in GEO number GSE294562. Data from single cell RNA sequencing data of lung vs mammary gland tissues can be found in GSE287197. Datasets generated during this study will be available in the Data archiving platform Figshare through The Francis Crick Institute.

## Statement of significance

In this work we propose a new radio therapy-sensitizing combination with the amino acid degrader Cyst(e)inase to treat breast cancer metastasis to the lung, tackling the specific dependence of metastatic cells on cysteine and cystine.
