# Supplementary material for: Targeting cystine addiction to treat breast cancer lung metastasis
Source: Cancer Res. Author manuscript; Available in PMC 2026 Aug 18. (PMC7619373; doi:10.1158/0008-5472.CAN-25-3883)
Supplement: Supplementary file 1 [file EMS217500-supplement-Supplementary_Fig__S1.docx]

**Supplementary Figure 1 (related to Figure 1).**

(**A**) and (**B**) Metabolite levels in MGTs, lung metastatic lesions, metastasis surrounding lung tissue, and normal lungs. Relative abundance is normalised to internal standard [U-13C,15N]valine and dry weight (*n* = 6-7 mice per group). Data are MEAN ± SD. * *p*<0.05 determined by *t* test. (**C**) and (**D**) Single ion images of glutamine and glutamate detected by DESI-MSI in consecutive slices from normal lungs, MGT and lungs from Myc310-MGT- and LM1-MGT-bearing mice infused with [U-^13^C]glutamine for 3 h. Consecutive H&E images and quantification of the ion intensities per pixel in the indicated regions are also shown. * p<0.05 determined by *t* test. (**E**) Pseudobulk reactome profile pathways downregulated in lung metastasis tumour cells *vs* primary tumour cells from LM1-MGT-bearing mice determined by single-cell RNA sequencing performed on lungs and primary tumours. *n* = 3 mice per group.

**Supplementary Figure 2 (related to Figure 2).**

(**A**) Metabolite levels in the serum of either LM1-MGT-bearing mice or control mice. Relative abundance is normalised to internal standard [U-^13^C,15N]valine (*n* = 6-7 mice per group). Data are MEAN ± SD. * *p*<0.05 determined by *t* test. (**B**) Schematics representing the *in vivo* and *ex vivo* models used to compare metastatic *vs* primary tumours. (**C**) Representative H&E images of lung sections from mice bearing orthotopic tumours induced by injecting either MYAZ16.e, Myc310 or LM1 cells into the mammary fat pad. (*n =* 8 mice per group) (**D**) mRNA levels of the indicated genes in Myc310 MGTs and LM1 MGTs relative to MYAZ16.1e MGTs measured by RT-qPCR, with beta-actin expression used for normalization. A representative experiment of three independent experiments with three replicates each is shown. MEAN of 2^(-ΔΔCt)^ is represented in the value of the heatmap. * *p*<0.05 determined by *t* test.

**Supplementary Figure 3 (related to Figure 3 and 4).**

(**A**) Selected pathways from those significantly enriched (*p*.adjusted<0.01, Benjamini-Hochberg adjustment) under Gene Set Enrichment Analyses (GSEA), following bulk mRNA sequencing and differential expression analysis of Myc310, LM (LM1, LM2, LM3) and LM-LM (LM1-LM, LM2-LM, LM3-LM) cell lines compared to MYAZ cell lines (MYAZ16.1e, MYAZ12.3i, MYAZ6.6g). Three highest and three lowest Normalised Enrichment Scores (NES) per comparison group were selected. Groups may have more than three points in the plot if a pathway in another group’s highest/lowest three was also significantly enriched (*p*.adjusted<0.01). Colour scaled by Normalised Enrichment Score (NES). Results grouped by gene set lists: GO consortium Biological Processes (GO_BP); Reactome ontologies; and entries belonging to the C2 ontology group in the MSigDB Collection (MSigDB_Curated). (**B**) Select Transcription Factor Targets (TFT) ontologies (GTRD subset) significantly enriched (*p*.adjusted<0.05, BH adjustment) under GSEA performed for comparisons of starved Myc310, LM (as above) or LM-LM (as above) cell lines compared to starved MYAZ cell lines (as above). Selection as in **A**. Colour scaled by NES. The experiment was performed using three biological replicates and three technical replicates. (**C**) Quantification of western blots from Figure 3F.

**Supplementary Figure 4 (related to Figure 4).**

(**A**) The confluence of indicated cell lines cultured in the absence of indicated amino acids measured by Incucyte®. (**B**) Cell number obtained by Scepter®. (**C**) Tumouroid growth of cells seeded in plates coated with Cultrex^TM^, under either cysteine/cystine (Cys/CySS) deprivation or control conditions applied 6h after seeding (at time 0). Confluence was analysed and images were taken with Incucyte®. Zoomed in images of tumoroids are shown below. (**D**) Imidazole ketone erastin IC50 curve and IC50 values using confluence data measured by Incucyte®. (**E**) Quantification of CellROX staining using ImageJ after 21h, corresponding to images in Fig. 4E * p<0.05 determined by *t* test. (**F**) Quantification of western blots from figure 4H. (**G**) Cell confluence measured by Incucyte® under low cysteine/cystine conditions (4 μM of each) either in the presence of absence of either 2 μM ferrostatin or 0.1 μM liproxtatin at 72h.

In all cases, a representative of three independent experiments is shown. Data are MEAN ± SD of technical replicates.

**Supplementary Figure 5 (related to Figure 5).**

(**A**) - (**C**) Metabolite levels (in **A** and **B**) and fractional enrichments (in **C**) in the indicated cell lines incubated with [U-^13^C]serine under either low Cys/CySS (4 μM of cystine and 4 μM of cysteine) or control conditions for 52h. Relative abundance was normalised to internal standard [U-^13^C,^15^N]valine and protein concentration. A representative of three independent experiments with three replicates each is shown. Data are MEAN ± SD of technical replicates. * *p*<0.05 determined by *t* test. (**D**) Differentially expressed genes from bulk mRNA sequencing experiment comparing cysteine/cystine-deprived (for 6h) vs control conditions in MYAZ16.1e, Myc310, LM1, and LM1-LM cells. Colour scale is Log2 Fold Change. * *p* <0.05 (IHW adjustment). (**E**) Selected significantly enriched pathways (*p*.adjusted<0.01) following comparison of cysteine/cystine-deprived *vs* non deprived MYAZ, Myc310, LM and LM_LM cell lines. Pathways were tested across a collection of gene sets: GO consortium Biological Processes (GO_BP); and entries belonging to the C2 otology group in the MSigDB Collection (MSigDB_Curated). Selection criteria for plotting as in S3A. Colour scaled by NES. The experiment was performed using three biological replicates and three technical replicates. (**F**) Quantification of western blots from Figure 4D. (**G**) Tumouroid growth of cells seeded in plates coated with Cultrex^TM^, under either cysteine/cystine (Cys/CySS) deprivation or control conditions applied 6h after seeding (at time 0). Confluence was analysed and images were taken with Incucyte®.

In all cases, a representative of three independent experiments is shown. Data are MEAN ± SD of technical replicates.

**Supplementary Figure 6 (related to Figure 6).**

(**A**) Weights of FVB/NJ mice kept on either control or cysteine/cystine free diet for 2 weeks before tumour induction by injection of LM1 cells into the mammary fat pad, and until the endpoint of 4 weeks after the injection (*n* = 5 mice per group). Data are MEAN ± SD. (**B**) and (**C**) Cysteine and cystine levels in the serum of mice from. ns = not significantly different with *p* < 0.05 determined by *t* test. Relative abundance was normalised to internal standard [U-13C,15N]valine (*n* = 5 mice per group). Data are MEAN ± SD. (**D**) Tumour growth from the experiment in **A**. Data are MEAN ± SD. * *p*<0.05 determined by *t* test. (**E**) Area of metastatic lesions in the lungs from the experiment in **A** measured from three serial sections each (separated by 100 μM). Data are MEAN ± SD. (**F**) CT images were taken with a Quantum X2 microCT at the endpoint of the experiment described in Figure 6C-D, and 3D stack was generated using AnalyzeDirect software. (**G**) Cysteine levels obtained from tissues of mice at the endpoind of the experiment described in Figure 6C-D. Relative abundance is normalised to internal standard [U-^13^C,1^5^N]valine and dry weight. Data are MEAN ± SD. * *p*<0.05 determined by *t* test. (**H**) Metastatic volume measured with microCT scans and AnalyzeDirect software at the endpoint of an experiment where FVB/NJ mice were injected with LM1 cells in the mammary fat pad, and 3 rounds of radiation (different doses specified) targeted to the lungs were performed in the third week after the injection (*n* = 5 mice per group). Data are MEAN ± SD. *p* value calculated using *t* test. (**I**) Mice weights for the experiment in **H**. Data are MEAN ± SD. (**J**) Tumour growth in the experiment described in Figure 6H-I. Data are MEAN ± SD. ns = not significantly different with *p* < 0.05 determined by *t* test. (**K**) Total metastatic volume in FBV/NJ mice with the orthotopic injection of LM1 cells treated with either 100 mg/kg of Cyst(e)inase or lung-targeted 4Gy irradiation, or both treatments (*n* = 4 mice per group). Data are MEAN ± SD. * *p*<0.05 determined by *t* test. (**L**) and (**M**) Cysteine and cystine levels in the serum for the experiment in Figure 6H-I. Data are MEAN ± SD. * *p*<0.05 determined by *t* test. (**N**) Mice weights for the experiment in Figure 6H-I. Data are MEAN ± SD.

**Supplementary Figure 7 (related to Figure 6).**

(**a**) CT images were taken with a Quantum X2 microCT at the endpoint of the experiment described in Figure 6h-i, and 3D stack was generated using AnalyzeDirect software.

| **REACTOME PATHWAYS UPREGULATED Cluser *Metastatic cells in the lung* vs *Mammary gland tumour cells,* using Pseudobulk analysis** | | | | |
| --- | --- | --- | --- | --- |
| **ID** | **Description** | **Gene Ratio** | **pvalue** | **Gene name** |
| R-MMU-202733 | Cell surface interactions at the vascular wall | 21/470 | 1.8019E-07 | Selp/Gm5150/Fcer1g/Trem1/Src/Inpp5d/  Sell/Ceacam1/Itgax/Sirpa/Slc7a11/Tek/Thbd/  Jaml/Pecam1/Itgb2/Cd84/Esam/Grb14/Gp6/  Cd47 |
| R-MMU-6783589 | Interleukin-6 family signaling | 8/470 | 4.2777E-06 | Clcf1/Osmr/Jak2/Socs3/Lifr/Osm/Il6ra/Il31 |
| R-MMU-5668599 | RHO GTPases Activate NADPH Oxidases | 8/470 | 2.0676E-05 | Ncf2/Ncf4/Cybb/S100a8/Cyba/S100a9/Ncf1/  Rac2 |
| R-MMU-76002 | Platelet activation, signaling and aggregation | 29/470 | 2.8943E-05 | Plek/Selp/Fcer1g/Syk/Dgkg/Lcp2/Ppbp/Src/  Timp3/Serping1/Cd36/Prkcg/Vwf/Vegfd/Trf/  Clec1b/Pik3r3/Pecam1/Rac2/Rap1b/Pdgfa/  Dgkh/Pik3cg/Dgka/Serpina3f/Tmsb4x/Gp6/  Actn1/F5 |
| R-MMU-6788467 | IL-6-type cytokine receptor ligand interactions | 6/470 | 4.5401E-05 | Clcf1/Osmr/Jak2/Lifr/Osm/Il31 |
| R-MMU-5683826 | Surfactant metabolism | 7/470 | 6.927E-05 | Csf2ra/Sftpb/Sftpd/Napsa/Slc34a2/Gata6/  Ctsh |
| R-MMU-391160 | Signal regulatory protein family interactions | 5/470 | 8.3617E-05 | Tyrobp/Gm5150/Sftpd/Sirpa/Cd47 |
| R-MMU-3299685 | Detoxification of Reactive Oxygen Species | 8/470 | 0.00025561 | Ncf2/Ncf4/Cybb/Cyba/Gpx3/Gsr/Ncf1/  Sod3 |
| R-MMU-114604 | GPVI-mediated activation cascade | 8/470 | 0.00031895 | Fcer1g/Syk/Lcp2/Clec1b/Pik3r3/Rac2/  Pik3cg/Gp6 |
| R-MMU-449147 | Signaling by Interleukins | 27/470 | 0.0004329 | Clcf1/Csf3r/Syk/Csf2ra/Il1r2/Osmr/Inpp5d/  Jak2/Ager/Socs3/Hck/Il10ra/Pik3r3/Il1rn/  Il1b/Lifr/Osm/Il4ra/Il13ra1/Csf1r/Il6ra/Il5ra/  Irak3/Stx3/Nfkb2/Tslp/Il31 |
| R-MMU-1222556 | ROS and RNS production in phagocytes | 8/470 | 0.00048405 | Ncf2/Ncf4/Cybb/Cyba/Slc11a1/Ncf1/Atp6v0a2/  Rac2 |
| R-MMU-76005 | Response to elevated platelet cytosolic Ca2+ | 16/470 | 0.00058083 | Plek/Selp/Ppbp/Timp3/Serping1/Cd36/Prkcg/  Vwf/Vegfd/Trf/Pecam1/Pdgfa/Serpina3f/  Tmsb4x/Actn1/F5 |

**Supplementary Table 1 (Related to Figure 1)**

List of pathways significantly upregulated in *lung metastatic cells in comparison with* primary tumour cells in LM1-MGT-bearing mice determined by single-cell RNA sequencing performed on lungs and primary tumours. Upregulated pseudobulk Reactome pathways are shown. *n* = 3 mice per group.

| **REACTOME PATHWAYS DOWNREGUTATED Cluser *Metastatic cells in the lung* vs *Mammary gland tumour cells,* using Pseudobulk analysis** | | | | |
| --- | --- | --- | --- | --- |
| **ID** | **Description** | **Gene Ratio** | **pvalue** | **Gene name** |
| R-MMU-1799339 | SRP-dependent cotranslational protein targeting to membrane | 29/196 | 3.3416E-24 | Rps14/Rpl32/Rps16/Rps15/Rpl5/Rps3/Rps25/  Rps5/Rpl27a/Rpl18a/Rps23/Rpl18/Rps19/  Rps9/Rps13/Rpl13/Rpl26/Rps4x/Rpl12/Rpl7/  Rpl19/Rpl23a/Rps10/Rpl27/Rpl11/Rps24/Rpl28/  Rpl22/Rpl23 |
| R-MMU-975956 | Nonsense Mediated Decay (NMD) independent of the Exon Junction Complex (EJC) | 29/196 | 3.5233E-24 | Rps14/Rpl32/Rps16/Rps15/Rpl5/Rps3/  Rps25/Rps5/Rpl27a/Rpl18a/Rps23/Rpl18/  Rps19/Rps9/Rps13/Rpl13/Rpl26/Rps4x/  Rpl12/Rpl7/Rpl19/Rpl23a/Rps10/Rpl27/  Rpl11/Rps24/Rpl28/Rpl22/Rpl23 |
| R-MMU-72689 | Formation of a pool of free 40S subunits | 29/196 | 2.7328E-23 | Rps14/Rpl32/Rps16/Rps15/Rpl5/Rps3/  Rps25/Rps5/Rpl27a/Rpl18a/Rps23/Rpl18/  Rps19/Rps9/Rps13/Rpl13/Rpl26/Rps4x/  Rpl12/Rpl7/Rpl19/Rpl23a/Rps10/Rpl27/  Rpl11/Rps24/Rpl28/Rpl22/Rpl23 |
| R-MMU-156827 | L13a-mediated translational silencing of Ceruloplasmin expression | 29/196 | 4.752E-22 | Rps14/Rpl32/Rps16/Rps15/Rpl5/Rps3/  Rps25/Rps5/Rpl27a/Rpl18a/Rps23/  Rpl18/Rps19/Rps9/Rps13/Rpl13/Rpl26/  Rps4x/Rpl12/Rpl7/Rpl19/Rpl23a/Rps10/  Rpl27/Rpl11/Rps24/Rpl28/Rpl22/Rpl23 |
| R-MMU-72706 | GTP hydrolysis and joining of the 60S ribosomal subunit | 29/196 | 5.1013E-22 | Rps14/Rpl32/Rps16/Rps15/Rpl5/Rps3/  Rps25/Rps5/Rpl27a/Rpl18a/Rps23/  Rpl18/Rps19/Rps9/Rps13/Rpl13/Rpl26/  Rps4x/Rpl12/Rpl7/Rpl19/Rpl23a/Rps10/  Rpl27/Rpl11/Rps24/Rpl28/Rpl22/Rpl23 |
| R-MMU-927802 | Nonsense-Mediated Decay (NMD) | 29/196 | 6.4958E-22 | Rps14/Rpl32/Rps16/Rps15/Rpl5/Rps3/  Rps25/Rps5/Rpl27a/Rpl18a/Rps23/  Rpl18/Rps19/Rps9/Rps13/Rpl13/Rpl26/  Rps4x/Rpl12/Rpl7/Rpl19/Rpl23a/Rps10/  Rpl27/Rpl11/Rps24/Rpl28/Rpl22/Rpl23 |
| R-MMU-975957 | Nonsense Mediated Decay (NMD) enhanced by the Exon Junction Complex (EJC) | 29/196 | 6.4958E-22 | Rps14/Rpl32/Rps16/Rps15/Rpl5/Rps3/Rps25/  Rps5/Rpl27a/Rpl18a/Rps23/Rpl18/Rps19/  Rps9/Rps13/Rpl13/Rpl26/Rps4x/Rpl12/Rpl7/  Rpl19/Rpl23a/Rps10/Rpl27/Rpl11/Rps24/  Rpl28/Rpl22/Rpl23 |
| R-MMU-72613 | Eukaryotic Translation Initiation | 29/196 | 2.0286E-21 | Rps14/Rpl32/Rps16/Rps15/Rpl5/Rps3/  Rps25/Rps5/Rpl27a/Rpl18a/Rps23/  Rpl18/Rps19/Rps9/Rps13/Rpl13/Rpl26/  Rps4x/Rpl12/Rpl7/Rpl19/Rpl23a/Rps10/  Rpl27/Rpl11/Rps24/Rpl28/Rpl22/Rpl23 |
| R-MMU-72737 | Cap-dependent Translation Initiation | 29/196 | 2.0286E-21 | Rps14/Rpl32/Rps16/Rps15/Rpl5/Rps3/  Rps25/Rps5/Rpl27a/Rpl18a/Rps23/Rpl18/  Rps19/Rps9/Rps13/Rpl13/Rpl26/Rps4x/  Rpl12/Rpl7/Rpl19/Rpl23a/Rps10/Rpl27/  Rpl11/Rps24/Rpl28/Rpl22/Rpl23 |
| R-MMU-72766 | Translation | 33/196 | 4.9254E-17 | Rps14/Rpl32/Mrps35/Rps16/Rps15/  Rpl5/Rps3/Rps25/Rps5/Rpl27a/  Rpl18a/Rps23/Rpl18/Rps19/Rps9/  Rps13/Rpl13/Mrpl15/Rpl26/Rps4x/  Rpl12/Rpl7/Rpl19/Rpl23a/Rps10/Rpl27/  Eef1g/Rpl11/Rps24/Rpl28/Mrpl45/  Rpl22/Rpl23 |
| R-MMU-6791226 | Major pathway of rRNA processing in the nucleolus and cytosol | 29/196 | 1.8831E-16 | Rps14/Rpl32/Rps16/Rps15/Rpl5/Rps3/  Rps25/Rps5/Rpl27a/Rpl18a/Rps23/  Rpl18/Rps19/Rps9/Rps13/Rpl13/Rpl26/  Rps4x/Rpl12/Rpl7/Rpl19/Rpl23a/Rps10/  Rpl27/Rpl11/Rps24/Rpl28/Rpl22/Rpl23 |
| R-MMU-72312 | rRNA processing | 29/196 | 1.8831E-16 | Rps14/Rpl32/Rps16/Rps15/Rpl5/  Rps3/Rps25/Rps5/Rpl27a/Rpl18a/Rps23/  Rpl18/Rps19/Rps9/Rps13/Rpl13/Rpl26/  Rps4x/Rpl12/Rpl7/Rpl19/Rpl23a/Rps10/  Rpl27/Rpl11/Rps24/Rpl28/Rpl22/Rpl23 |
| R-MMU-8868773 | rRNA processing in the nucleus and cytosol | 29/196 | 1.8831E-16 | Rps14/Rpl32/Rps16/Rps15/Rpl5/Rps3/  Rps25/Rps5/Rpl27a/Rpl18a/Rps23/Rpl18/  Rps19/Rps9/Rps13/Rpl13/Rpl26/Rps4x/  Rpl12/Rpl7/Rpl19/Rpl23a/Rps10/Rpl27/  Rpl11/Rps24/Rpl28/Rpl22/Rpl23 |
| R-MMU-72695 | Formation of the ternary complex, and subsequently, the 43S complex | 13/196 | 1.3693E-09 | Rps14/Rps16/Rps15/Rps3/Rps25/  Rps5/Rps23/Rps19/Rps9/Rps13/Rps4x/  Rps10/Rps24 |
| R-MMU-72649 | Translation initiation complex formation | 13/196 | 7.2156E-09 | Rps14/Rps16/Rps15/Rps3/Rps25/  Rps5/Rps23/Rps19/Rps9/Rps13/Rps4x/  Rps10/Rps24 |
| R-MMU-72702 | Ribosomal scanning and start codon recognition | 13/196 | 7.2156E-09 | Rps14/Rps16/Rps15/Rps3/Rps25/  Rps5/Rps23/Rps19/Rps9/Rps13/Rps4x/  Rps10/Rps24 |
| R-MMU-72662 | Activation of the mRNA upon binding of the cap-binding complex and eIFs, and subsequent binding to 43S | 13/196 | 8.5846E-09 | Rps14/Rps16/Rps15/Rps3/Rps25/Rps5/  Rps23/Rps19/Rps9/Rps13/Rps4x/  Rps10/Rps24 |

**Supplementary Table 2 (Related to Figure 1)**

List of significantly downregulated pathways in *lung metastatic cells in comparison with* primary tumour cells in LM1-MGT-bearing mice determined by single-cell RNA sequencing performed on lungs and primary tumours. Downregulated pseudobulk Reactome pathways are shown. *n* = 3 mice per group.
